# Chlorhexidine vs Povidone-Iodine and Incidence of Catheter-Related Infections: A Systematic Review and Meta-Analysis

**DOI:** 10.1001/jamanetworkopen.2025.58954

**Published:** 2026-02-12

**Authors:** Bertrand Drugeon, Gabor Mihala, Jessica Schults, Benjamin Bigaud, Jérémy Guenezan, Guillaume Batiot, Natalie Barker, Nicolas Marjanovic, Niccolò Buetti, Olivier Mimoz

**Affiliations:** 1 Service des Urgences Adultes–SAS 86-Centre 15, Centre Hospitalier Universitaire de Poitiers, Poitiers, France; 2 Institut National de la Santé et de la Recherche Médicale, U1070, Pharmacologie des Agents Anti-Infectieux et Resistance, Poitiers, France; 3 Alliance for Vascular Access Teaching and Research, Griffith University, Nathan, Australia; 4 School of Nursing, Midwifery and Social Work, University of Queensland, Brisbane, Queensland, Australia; 5 Faculté de Médecine et de Pharmacie, Université de Poitiers, Poitiers, France; 6 Centre for Health Services Research, Faculty of Health, Medicine and Behavioural Sciences, The University of Queensland, Woolloongabba, Queensland, Australia; 7 Herston Infectious Diseases Institute, Metro North Health, Brisbane, Queensland, Australia; 8 Herston Health Sciences Library, The University of Queensland, Herston, Queensland, Australia; 9 Institut National de la Santé et de la Recherche Médicale Centre d'Investigation Clinique 1402, Centre Hospitalier Universitaire de Poitiers, Poitiers, France; 10 Infection Control Program and World Health Organization Collaborating Centre, Faculty of Medicine, Geneva University Hospitals, Geneva, Switzerland; 11 Institut National de la Santé et de la Recherche Médicale, Infection, Antimicrobials, Modelling, Evolution, Université Paris-Cité, Paris, France; 12 Centre for Clinical Research, University of Queensland, Brisbane, Queensland, Australia

## Abstract

**Importance:**

Intravascular catheters are essential in health care but remain a major source of health care–associated infections. Optimal skin antisepsis before insertion is key, yet the most effective antiseptic agent, concentration, and formulation remain uncertain.

**Objective:**

To determine the concentration and formulation of chlorhexidine gluconate (CHG) or povidone-iodine (PVI) associated with the lowest incidence of catheter-related infections (CRIs).

**Data Sources:**

PubMed, EMBASE, Cochrane Central, Scopus, Web of Science, and CINAHL were searched through January 7, 2025, without restrictions. Trial registries and reference lists of relevant studies and guidelines were reviewed.

**Study Selection:**

Randomized clinical trials (RCTs) comparing CHG- or PVI-based skin antisepsis before insertion of intravascular catheters were eligible if they reported at least 1 CRI outcome (catheter-related bloodstream infection [CRBSI], catheter tip colonization, or local infection). Two independent reviewers screened titles, abstracts, and full texts.

**Data Extraction and Synthesis:**

Data were extracted independently by 2 reviewers following Preferred Reporting Items for Systematic Reviews and Meta-Analyses (PRISMA) guidelines. Risk of bias was assessed with the Cochrane risk of bias 2 tool. A random-effects network meta-analysis (NMA) was performed to estimate relative risks (RRs) with 95% CIs.

**Main Outcomes and Measures:**

The primary outcomes were incidence of CRBSIs, catheter tip colonization, and local infections associated with CHG or PVI formulations.

**Results:**

Sixteen RCTs (7803 patients; 11 985 catheters) met inclusion criteria. When compared with aqueous formulations, alcohol-based formulations were consistently associated with lower infections rates, with isopropyl alcohol being superior to ethanol. Compared with alcoholic PVI, alcoholic CHG was associated with lower CRBSIs (RR, 0.70 [95% CI, 0.45 to 1.08]), catheter tip colonizations (RR, 0.42 [95% CI, 0.37 to 0.48]), and local infections (RR, 0.40 [95% CI, 0.23 to 0.70]). High concentration CHG (1% or higher) further lowered CRBSIs (RR, 0.31 [95% CI, 0.19 to 0.52]) and colonization (RR, 0.36 [95% CI, 0.30 to 0.42]) compared with lower concentrations. Local adverse events were uncommon, slightly more frequent with alcohol-based formulations, and similar between CHG and PVI.

**Conclusions and Relevance:**

In this NMA of RCTs, high concentration CHG in isopropyl alcohol was associated with the lowest incidence of CRIs. These results suggest that alcoholic PVI and aqueous formulations should be reserved for situations in which CHG or alcohol cannot be used.

## Key Points

**Question:**

What concentration and formulation of chlorhexidine or povidone-iodine is associated with the lowest incidence of catheter-related infections (catheter-related bloodstream infections, catheter tip colonization, or local infections)?

**Findings:**

In this network meta-analysis and systematic review including 11 985 catheters from 16 randomized trials, alcohol-based rather than aqueous-based formulations, isopropyl alcohol rather than ethanol, chlorhexidine-based rather than povidone-iodine formulations, and higher (1% or higher) rather than lower concentrations of chlorhexidine for skin preparation were associated with lower infection rates.

**Meaning:**

These findings suggest that high concentration chlorhexidine in isopropyl alcohol should be recommended as the first-line skin antiseptic before intravascular catheter insertion.

## Teaser

This systematic review and meta-analysis examines the concentration and formulation of chorhexidine gluconate and providone-iodine that are associated with the lowest incidence of intravascular catheter-related infections.

## Introduction

Intravascular catheters are essential components of modern health care, enabling the administration of fluids, medications, blood products, and parenteral nutrition.^1^ In hospital, their use is ubiquitous, with billions of devices sold worldwide each year.^2,3^ Despite their essential role in patient care, these devices carry significant risks, making their safe use a priority in clinical practice.^4,5^

Catheter-related bloodstream infections (CRBSIs) are laboratory-confirmed bloodstream infections originating from intravascular catheters and represent the most serious catheter-related complication, ranking among the leading causes of hospital-acquired infections. Unlike central line–associated bloodstream infection (CLABSI), which is a surveillance definition, CRBSI requires microbiological confirmation of catheter origin. In the US, central venous catheters (CVCs) account for approximately 80 000 bloodstream infections annually in intensive care units, with mortality rates of 12% to 25%.^6^ Although peripheral intravenous catheters (PIVCs) carry a lower individual risk, their widespread use makes them a significant concern.^6,7^ CRBSIs increase morbidity, prolong hospital stays, raise health care costs, and contribute to antibiotic overuse.^8,9,10^

Catheter-related infections (CRIs) encompass a spectrum of infectious complications associated with intravascular devices, including CRBSIs, catheter tip colonization, and local insertion-site infections. These events arise from microbial contamination of the insertion site or catheter surface and represent a major contributor to device failure and patient morbidity. Skin disinfection prior to intravascular catheter insertion is the key preventive measure of CRIs, aiming to reduce microbial load at the insertion site.^11,12^ Chlorhexidine gluconate (CHG) and povidone-iodine (PVI), both in aqueous or alcoholic formulations, are the most widely used skin disinfectants. Key differences include a broader spectrum of activity, particularly against Gram-negative bacteria, shorter duration of efficacy,^13^ and longer drying time with PVI.^14^ High CHG (1% or higher) concentrations enhance antimicrobial activity, particularly against Gram-negative bacteria,^14,15^ and may prolong bactericidal effects compared with low concentrations. Alcohol is rapidly bactericidal and enhances membrane penetration and disrupts biofilms, thereby strengthening the activity of CHG and PVI^14,15,16,17,18,19,20^ and shortening PVI drying time. Previous comparisons have several limitations, leaving gaps in knowledge on the most effective strategies for preventing CRIs and contributing to variability in practices. They often compare CHG and PVI, without considering CHG concentration, formulation type (aqueous or alcoholic), and alcohol type (isopropyl alcohol [IPA] or ethanol); they are often restricted to specific populations^21^ or to a single catheter type.^12,22,23,24^ We aimed to determine the most effective skin antiseptic strategy for preventing CRIs, accounting for CHG concentration, antiseptic formulation, alcohol type, catheter type, and specific populations.

## Methods

### Search Strategy and Selection Criteria

We performed a systematic review and network meta-analysis (NMA) guided by Cochrane methods^25^ and reported in accordance with the Preferred Reporting Items for Systematic Reviews and Meta-Analyses (PRISMA) reporting guideline.^26^ The protocol was registered with PROSPERO (CRD42025634996). Ethics committee approval and informed consent were not required because this study involved analysis of published aggregate data only and did not include individual patient data.

We included randomized controlled trials (RCTs) that compared skin disinfection with aqueous- or alcohol-based formulations of CHG or PVI prior to intravascular catheter insertion in the hospital, regardless of patients’ age, provided they reported at least 1 predefined outcome associated with CRIs. Skin disinfection was defined as the preparation of the skin prior to the insertion of any intravascular catheter using any application method (single-use applicators vs multi-use bottles and sterile gauzes). CHG concentrations of 1% or higher were considered high. Peripherally inserted central catheters (PICCs), midline catheters, PIVCs, CVCs, arterial catheters (ACs), dialysis catheters (DCs), umbilical catheters (UCs), and pulmonary arterial catheters (PACs) were eligible. Crossover trials were eligible for inclusion. Conference abstracts were eligible if they provided extractable outcome data relevant to CRIs (eg, event counts and denominators for CRBSI, catheter tip colonization or local infection, catheter type, and antiseptic formulation or concentration), could be confirmed as reporting unique datasets not duplicated elsewhere and passed basic data-quality checks (internal consistency, concordance with trial registries, and author queries when feasible). We excluded studies with another study design (eg, reviews, narrative reviews, meta-analyses, observational studies), reporting intraosseous catheters, implantable ports, urinary catheters, perineural catheters, drains, or other implantable medical devices; reporting CLABSIs alone; studies involving simulation models, animals, or healthy volunteers; assessing efficacy of these antiseptic solutions for maintenance purposes (eg, impregnated dressings, skin disinfection during dressing changes, or hub, and connector disinfection). Studies involving octenidine and olanexidine were excluded from the search strategy, because they are available in only a few countries.

A systematic search was performed in the electronic databases PubMed, EMBASE, the Cochrane Library, Scopus, Web of Science, and CINAHL using Medical Subject Headings (MeSH). The search strings are provided in eAppendix 1 in Supplement 1.

Search results were imported into EndNote and then Covidence, which automatically removed duplicates and facilitated screen and review. Title and abstract screening, as well as full-text eligibility assessment, were performed independently by pairs of reviewers, selected from a team of 4 (B.D., O.M., B.B., G.B.). The Covidence platform automatically assigned each reference to 2 independent reviewers. If both reviewers agreed, the study was either included or excluded accordingly. In case of disagreement, a third reviewer was assigned to assess the study blindly to previous evaluations and to decide whether to include the study. The list of excluded full-text articles is provided in eAppendix 2 in Supplement 1.

### Outcome Measures

The primary outcome was the incidence of CRIs (ie, CRBSIs, catheter tip colonizations, and local infections), each reported and analyzed as individual outcomes. CRBSIs were defined according to the European Centre for Disease Prevention and Control (ECDC) definitions.^27^ Catheter tip colonization was defined as either 15 or more colonies forming unit (CFU) of a pathogen using the roll-plate semiquantitative method, or 1000 or more CFU of a pathogen per mL using the quantitative broth dilution culture technique.^28,29^ Local infection was defined as purulent drainage at insertion site, pain, with or without body temperature of more than 38 °C.^30^ Secondary outcomes were time from catheter insertion to CRI, adverse events at skin insertion site or anaphylaxis, length of hospital stay (LOS), and all-cause in hospital mortality.

The unit of analysis varied across studies: some reported outcomes per patient, while others reported outcomes per catheter. For infection-related outcomes, data were extracted at the catheter level when directly reported, as this was the most common reporting unit across studies. No conversion of patient-level outcomes or rates expressed per catheter-days into device-level event counts was performed. For outcomes inherently linked to individual patients (eg, mortality, side effects), data were extracted at the patient level. When multiple catheters were inserted in the same patient, and no adjustment for clustering was provided, we assumed independence between devices, as individual-level clustering data were not available.

### Statistical Analysis

Data were extracted independently by 2 reviewers using a standardized data extraction form in Covidence. In the event of discrepancies, a third reviewer independently re-extracted the data to resolve disagreements. Extracted data included study and participant characteristics, intervention (antiseptic agent, concentration, formulation, application method), catheter characteristics (type, site), and outcome data for all prespecified measures.

Effect estimates for each pairwise comparison were expressed as relative risk (RR) for a dichotomous outcome and mean difference (MD) for a continuous outcome, both with 95% CIs. We performed a random-effects NMA to integrate direct and indirect evidence. For the NMA, we included all RCTs comparing CHG-based with PVI-based skin antisepsis. Each treatment arm was classified into a predefined node according to antiseptic agent (CHG vs PVI), CHG concentration (high [1% or higher vs low [lower than 1%]), formulation (alcohol-based vs aqueous-based), and type of alcohol (IPA vs ethanol). Each unique combination of these characteristics constituted a distinct intervention node. Head-to-head CHG–PVI comparisons from individual trials defined the edges between nodes. Risk of bias was assessed using the Cochrane risk of bias 2 tool.^31^ Preplanned subgroup analyses stratified results by patient population (adults vs infants), catheter type (peripheral vs central), and publication decade. Sensitivity analyses excluded studies at high risk of bias and included conventional pairwise meta-analyses. In exploratory analyses, we compared high vs low CHG concentrations, alcohol-based vs aqueous-based formulations, and ethanol-based vs IPA-based preparations, after pooling CHG-containing and PVI-containing preparations within each alcohol group. Heterogeneity was assessed using the *I^2^* statistic and τ^2^. Local network consistency was evaluated using node-splitting, which compares direct and indirect evidence for each comparison. Global inconsistency across the entire network was assessed using the design-by-treatment interaction model. Analyses were conducted in R version 4.2.1 (R Project for Statistical Computing) using the *metafor*,^32^ *Igraph*,^33^ and *netmeta*^34^ packages. Full statistical procedures, including data transformation, handling of multi-arm trials, continuity correction, Surface Under the Cumulative Ranking Curve (SUCRA), and method for assessing transitivity, consistency, and reporting bias, are detailed in eAppendix 3 in Supplement 1.

## Results

**Figure 1.**
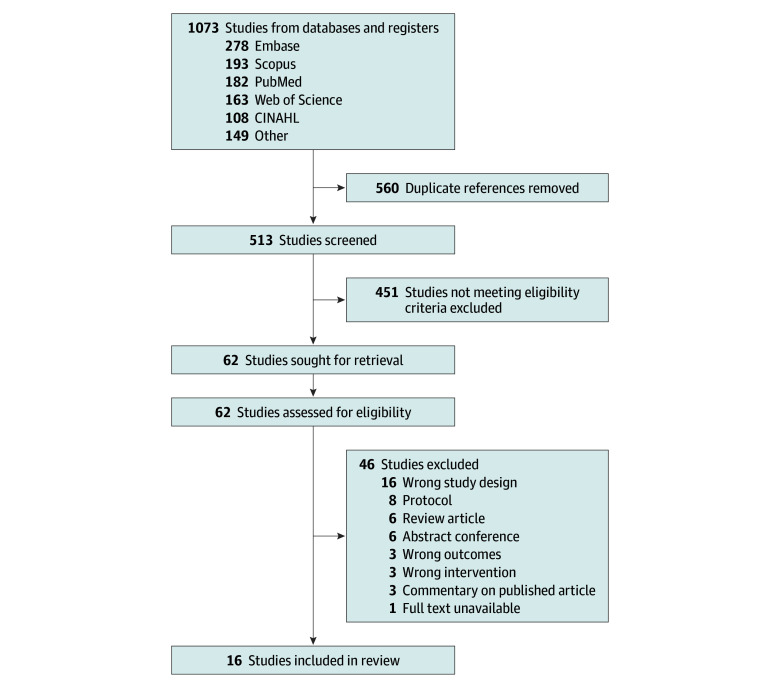
PRISMA Flow Diagram of Study Inclusion PRISMA indicates Preferred Reporting Items for Systematic Reviews and Meta-Analyses.

The search identified 1073 records. After removing 560 duplicates and excluding 451 ineligible records, 62 full texts were assessed for eligibility, and 16 studies^35,36,37,38,39,40,41,42,43,44,45,46,47,48,49,50^ were included, comprising 8154 patients and 12 420 catheters: 4396 CVCs (35%), 4111 arterial catheters (33%), 1848 PIVCs (15%), 618 DCs (5%), 200 umbilical catheters (2%), 98 PACs (0.8%), 62 introducer sheaths (0.5%), 48 PICCs (0.4%), and 1039 unspecified catheters (Figure 1). Catheter characteristics are outlined in the Table. Subgroups of 351 patients and catheters from 3 studies were excluded for alcohol rinsing of skin initially disinfected with PVI,^35^ for combining CHG and PVI in the same patients,^36^ or disinfecting the skin with IPA alone.^37^ After excluding nontarget comparator arms and uncultured catheters, 7803 patients and 11 985 catheters (6146 skin disinfections using CHG and 5839 using PVI) remained for analysis. Two studies were conducted in neonatal ICUs,^39,42^ 12 in adult ICUs,^35,36,37,41,43,44,45,46,47,48,49,50^ and 4 in emergency departments and wards.^35,36,38,40^ CHG was used at concentration ranging from 0.25% to 2%, and PVI from 5% to 10%, both in aqueous or alcoholic (ethanol or IPA) formulations. The combination of 0.25% CHG, 4% benzyl alcohol, and 0.025% benzalkonium chloride was considered as an aqueous formulation based on its low alcohol concentration. Mean (SD) catheter dwell time ranged from 1.7 (0.1) days for PIVCs to 9.6 (5.1) days for long-term devices. Assessment of transitivity and coherence showed no evidence of inconsistency for CRBSIs or local infections. For catheter tip colonization, the node-splitting analyses did not reveal significant local inconsistency, although the global inconsistency test indicated disagreement between designs (eAppendix 4 in Supplement 1).

**Table.** Characteristics of Included Studies^a^

Source	Country	Centers, No.	Setting	Study design	Intervention	Comparisons	Type of catheter	Catheter dwell time	Sample size	Outcomes
Atahan et al,^38^ 2012	Turkey	1	Surgical ward	RCT 2 groups	10% PVI vs 1.5% CHG + 15% cetrimide + EtOH	Aqueous PVI vs alcoholic CHG	CVC	Unknown	50 patients; 50 catheters	CRBSI; catheter tip colonization
Cobbett et al,^35^ 2000	Canada	1	Medical, surgical and obstetrical ward, ED and ICU	RCT 3 groups	10% PVI then 70% IPA vs 70% IPA then 10% PVI vs 0.5% CHG-70% IPA	Aqueous PVI then alcohol vs alcoholic PVI vs alcoholic CHG	PIVC	1.8 d (mean)	244 patients; 244 catheters	Catheter tip colonization; local infection
Garland et al,^39^ 2009	United States	5	NICU	Pilot RCT 2 groups	10% PVI vs 2% CHG	Aqueous PVI vs aqueous CHG	PICC	14.8 d (mean)	48 patients 48 catheters	Catheter tip colonization; CRBSI; adverse events
Guenezan et al,^40^ 2021	France	1	ED Medical wards	RCT 2 groups	5% PVI-69% EtOH vs 2% CHG-70% IPA	Alcoholic PVI vs alcoholic CHG	PIVC	1.6 d (median)	989 patients; 989 catheters	CRIs (local infection + catheter tip colonization + CRBSI); CRBSI; local infection; catheter tip colonization; adverse events
Humar et al,^41^ 2000	Canada	3	ICU	RCT 2 groups	10% PVI vs 0.5% CHG-IPA	Aqueous PVI vs alcoholic CHG	CVC	7.6 d (mean)	242 patients; 242 catheters	CRBSI; catheter tip colonization; local infection
Kieran et al,^42^ 2018	Ireland	2	NICU	RCT 2 groups	10% PVI vs 2% CHG-70% IPA	Aqueous PVI vs alcoholic CHG	Umbilical catheter PICC	9 d (median)	304 patients; 815 catheters	CRBSI; adverse events; late sepsis; thyroid dysfunction
Langgartner et al,^36^ 2004	Germany	1	Medical ward ICU	RCT 3 groups	10% PVI vs 0.5% CHG-70% IPA vs 0.5% CHG-70% IPA then 10% PVI	Aqueous PVI vs alcoholic CHG vs alcoholic CHG then aqueous PVI	CVC DC	13.7 d (mean)	119 patients; 140 catheters	Catheter tip colonization
Legras et al,^43^ 1997	France	1	ICU	RCT 2 groups	10% PVI vs 0.5% CHG-70% IPA	Aqueous PVI vs alcoholic CHG	CVC AC DC PAC	20.5 d (mean)	190 patients; 457 catheters	Catheter tip colonization; CRBSI; microbiological profile of catheter infections and bacteraemia’s; site-of-insertion analysis of CRI incidence
Maki et al,^37^ 1991	United States	1	ICU	RCT 3 groups	10% PVI vs 70% IPA vs 2% CHG	Aqueous PVI vs alcohol vs aqueous CHG	CVC AC	4.9 d (mean)	668 patients; 668 catheters	Catheter tip colonization; CRBSI; BSI due to the contamination of the hub/infusate
Maki et al,^44^ 2001	United States	1	ICU	RCT 2 groups	10% PVI vs 1% CHG-75% EtOH	Aqueous PVI vs alcoholic CHG	CVC PICC AC	Unknown	1039 catheters	Catheter tip colonization; CRBSI
Mimoz et al,^46^ 1996	France	1	ICU	RCT 2 groups	10% PVI vs 0.25% CHG-0.025% benzalkonium-4% benzyl alcohol	Aqueous PVI vs aqueous CHG	CVC AC	5.5 d (mean)	162 patients; 315 catheters	Catheter tip colonization; CRBSI; catheter-related sepsis; device-specific analyses; pathogen-specific analyses
Mimoz et al,^45^ 2007	France	1	ICU	RCT 2 groups	5% PVI-69% EtOH vs 0.25% CHG-0.025% benzalkonium-4% benzyl alcohol	Alcoholic PVI vs aqueous CHG	CVC	12 d (mean)	399 patients; 481 catheters	Catheter tip colonization; CRBSI; time to colonization; microorganism-specific colonization; site-specific analyses for colonization risk; safety/tolerability
Mimoz et al,^47^ 2015	France	11	ICU	RCT 2 groups	5% PVI-69% EtOH vs 2% CHG-70% IPA	Alcoholic PVI vs aqueous CHG	CVC AC DC	6 d (median)	2349 patients; 5159 catheters	Catheter-related infection (catheter tip colonization + CRBSI); catheter tip colonization; CRBSI; skin colonization; mortality; adverse events
Sheehan et al,^50^ 1993	Canada	1	ICU	RCT 2 groups	10% PVI vs 2% CHG	Aqueous PVI vs aqueous CHG	CVC AC PAC DC IS	Unknown	94 patients; 346 catheters	Catheter tip colonization; CRBSI
Vallés et al,^48^ 2008	Spain	1	ICU	RCT 3 groups	10% PVI vs 2% CHG vs 0.5% CHG-70% EtOH	Aqueous PVI vs aqueous CHG vs alcoholic CHG	CVC AC	7.4 d (mean)	329 patients; 631 catheters	Catheter tip colonization; CRBSI; systemic infection; microbiological profile of colonizing organisms
Yasuda et al,^49^ 2017	Japan	16	ICU	RCT 3 groups	10% PVI vs 0.5% CHG-79% EtOH vs 1% CHG-79% EtOH	Aqueous PVI vs alcoholic low CHG concentration vs alcoholic high CHG concentration	CVC AC	3.8 d (median)	796 patients; 796 catheters	Catheter tip colonization; CRBSI; length of stay; mortality; adverse events

Abbreviations: AC, arterial catheter; BSI, blood stream infection; CHG, chlorhexidine gluconate; CRBSI, catheter-related bloodstream infection; CVC, central venous catheter; DC, dialysis catheter; ED, emergency department; EtOH, ethanol; IS, introducer sheath; ICU, intensive care unit; IPA, isopropyl alcohol; NICU, neonatal intensive care unit; PAC, pulmonary artery catheter; PICC, peripheral inserted central catheter; PIVC, peripheral intravenous catheter; PVI, povidone-iodine; RCT, randomized controlled trial.

^a^ Antiseptics were in aqueous solution unless otherwise stated.

**Figure 2.**
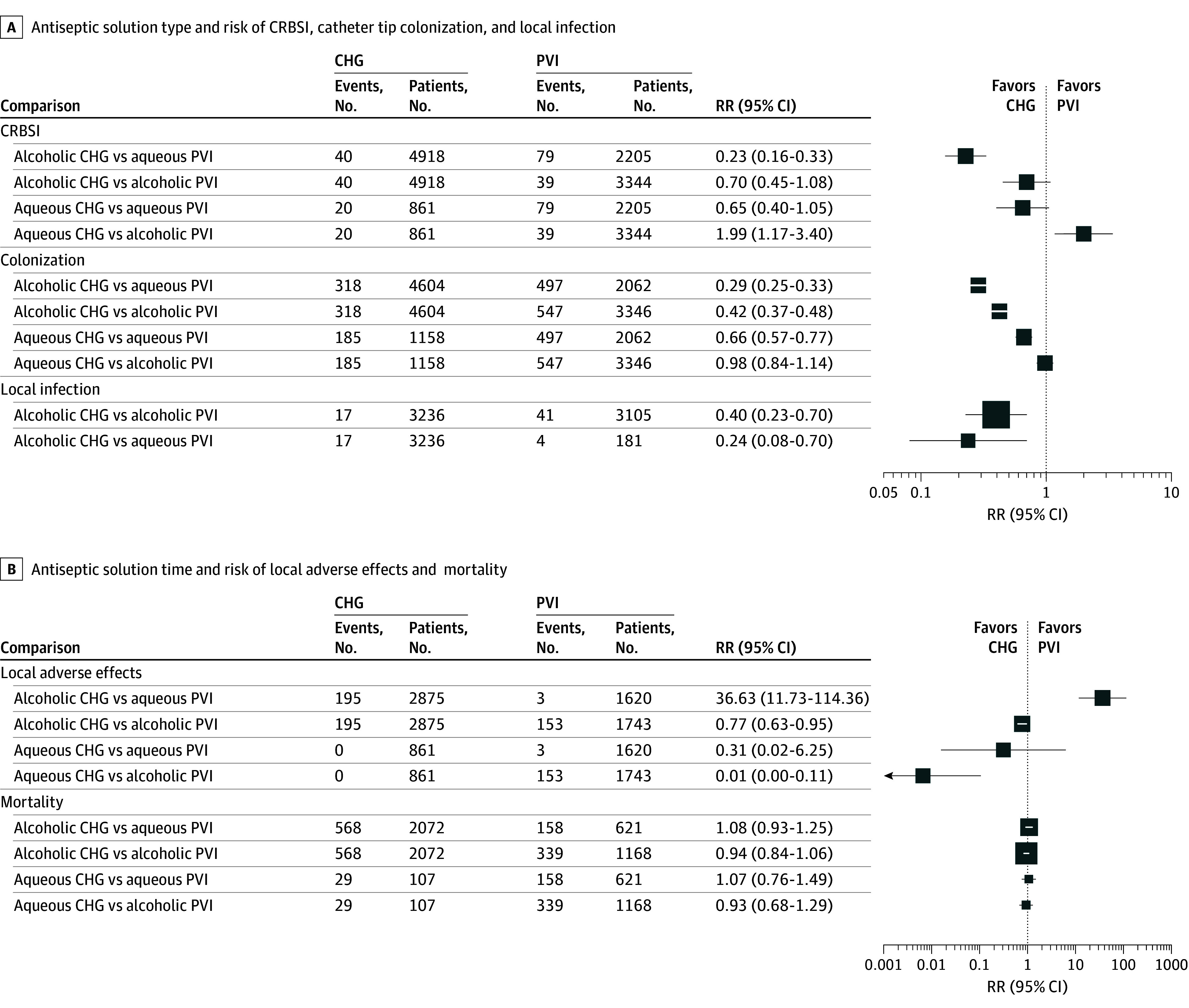
Forest Plots for Network Meta-Analysis of the Association Between Antiseptic Solution Type and CRBSI, Catheter Tip Colonization, and Local Infection and for Secondary Outcomes of Antiseptic Solutions and Mortality or Local Adverse Effects CHG, chlorhexidine gluconate; CRBSI, catheter-related bloodstream infection; PVI, povidone-iodine; RR, relative risk.

Thirteen studies examined the risk of CRBSIs by the antiseptic solution used.^37,38,39,40,41,42,43,44,45,46,47,48,49^ Alcoholic CHG was associated with a lower risk than aqueous or alcoholic PVI (eAppendix 5 in Supplement 1 and Figure 2). Aqueous CHG was associated with a lower risk than aqueous PVI and a higher risk than alcoholic PVI. Heterogeneity between studies was substantial (*I^2^* = 93.3%; 95% CI, 86.2 to 96.8%; τ^2^ = 0.73; 95% CI, 0.2 to 10.9; 95% prediction interval [PI], 0.03 to 14.3).

Fifteen studies examined the risk of catheter tip colonization by the antiseptic solution used.^35,36,37,38,39,40,41,43,44,45,46,47,48,49,50^ Alcoholic CHG was associated with a lower risk than aqueous or alcoholic PVI (eAppendix 5 in Supplement 1 and Figure 2). Aqueous CHG was associated with a lower risk than aqueous PVI and a similar risk to alcoholic PVI. Heterogeneity between studies was substantial (*I^2^* = 98.9%; 95% CI, 97.0 to 98.9%; τ^2^ = 0.28; 95% CI, 0.09 to 3.95; 95% PI, 0.08 to 3.48).

Only 3 studies examined the risk of local infections by antiseptic solution used.^40,41,47^ Alcoholic CHG was associated with a lower risk than alcoholic or aqueous PVI. No study involved aqueous CHG. The 95% PI ranged from 0.02 to 9.11.

**Figure 3.**
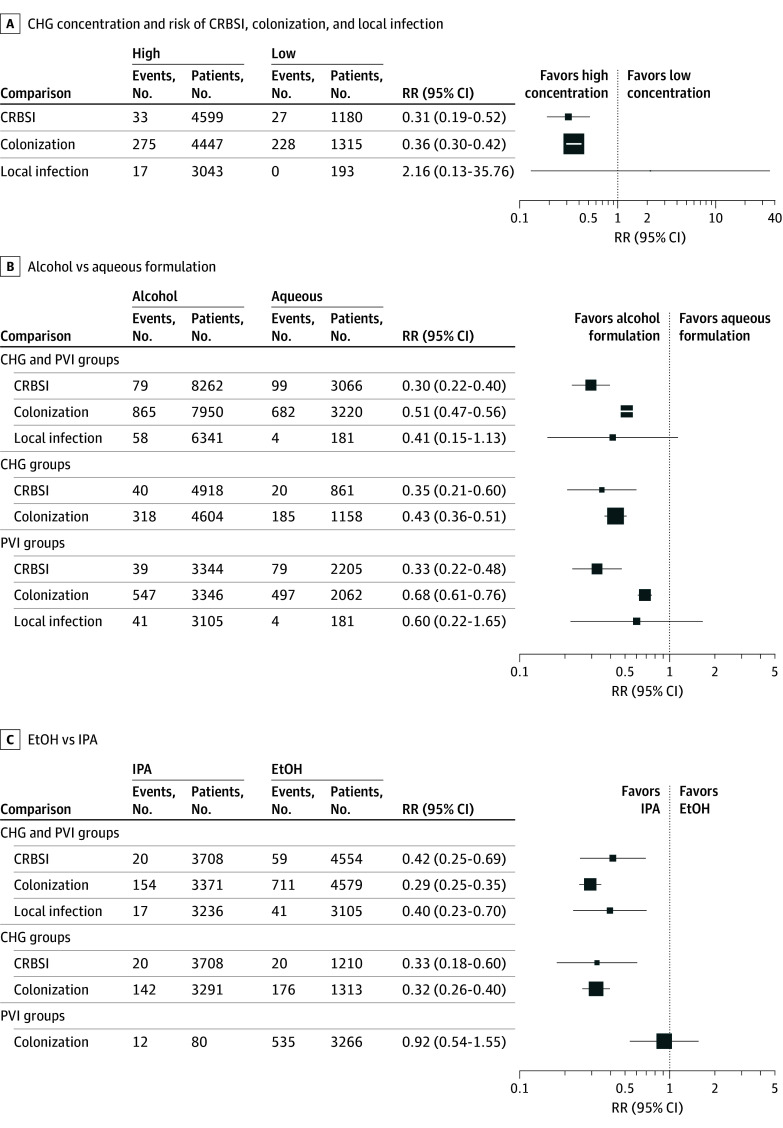
Exploratory Analysis of the Association Between Catheter-Related Infections and Chlorhexidine Gluconate Concentration (CHG) (High vs Low) and Formulations With Comparison of Alcohol vs Aqueous and EtOH vs IPA CRBSI, catheter-related bloodstream infection; EtOH, ethanol; IPA, isopropyl alcohol; PVI, povidone-iodine; RR, relative risk.

Rankograms and SUCRA values are provided in eAppendix 6 in Supplement 1. Sensitivity analyses confirmed the robustness of the main NMA findings: alcoholic CHG consistently outperformed other antiseptics in preventing CRIs; aqueous CHG was generally associated with a lower risk of CRIs than aqueous PVI and broadly comparable risk to alcoholic PVI. CHG was not associated with lower infectious risk than PVI in infants (eAppendix 7 in Supplement 1). Subgroup analyses by decade of publication showed that the superiority of CHG over PVI was consistent across periods. However, effect sizes tended to be greater in earlier trials and more precise in studies published after 2010 (eAppendix 8 in Supplement 1). In exploratory analysis, alcohol-based formulations were associated with lower catheter infectious risk than aqueous formulations. As were high concentration CHG vs low-concentration CHG and IPA-based solutions vs ethanol-based solutions (Figure 3).

No enrolled studies examined time to infection onset or reported cases of anaphylaxis associated with antiseptic use. Ten studies explored adverse events at the catheter insertion site according to the antiseptic solution used.^37,39,40,42,44,45,46,47,48,49^ Aqueous formulations were associated with lower complications than alcoholic formulations, while CHG and PVI had similar risk (Figure 2). Reported adverse events were exclusively minor cutaneous reactions, including erythema, mild irritation, and low-grade contact dermatitis, occasionally accompanied by localized edema or induration. Heterogeneity between studies was substantial (*I^2^* = 97.7%; 95% CI, 94.3% to 99.0%; τ^2^ = 11.35; 95% CI, 11.35 to 113.52).

Because LOS is influenced by multiple patient- and disease-related factors and displayed extreme heterogeneity (*I^2^* > 99%; τ^2^ = 442.52), the observed association with antiseptic type is not clinically meaningful (eAppendix 8 in Supplement 1). Five studies investigated the impact of antiseptic solution choice on all-cause in-hospital mortality (Figure 2).^42,43,47,48,49^ No difference was seen in the type of antiseptic solution used. Due to the wide confidence interval surrounding *I^2^* (0 to 84.7%), the degree of heterogeneity remains uncertain (τ^2^ = 0; 95% CI, 0 to 0.07). Study-level risk of bias across the Cochrane risk of bias tool 2 domains was generally low to moderate, and there was no evidence of small-study effects or reporting bias (eAppendix 9 in Supplement 1).

## Discussion

To our knowledge, we report the largest systematic review and NMA comparing skin disinfection with CHG or PVI for preventing CRIs, taking into account CHG concentration and antiseptic formulation, catheter type, and specific populations. With more than 11 500 catheters analyzed, we reported lower infection risk with alcohol-based than aqueous-based formulations and CHG than PVI. The superiority of CHG over PVI has persisted over time, despite the introduction of other preventive measures in bundles.^51^ Results were consistent across all sensitivity and subgroup analyses, except in infants, but this last observation was based on only 2 studies with a high risk of bias. Interestingly, higher CHG concentrations were associated with a lower risk of CRIs than lower concentrations, as was IPA compared with ethanol. These patterns further emphasize that the presence of alcohol is a major contributor to antiseptic performance and that differences observed between CHG and PVI in some comparisons likely reflect the formulation (alcoholic vs aqueous) rather than the active agent alone.

In the present study, high concentration CHG was associated with a lower risk of CRIs than low concentrations, a topic that is less frequently assessed. In 1 NMA of 5 studies enrolling 2815 CVCs and ACs in ICU patients, the hierarchy of efficacy in reducing CRBSI was 1% alcoholic CHG, 0.5% alcoholic CHG, 2% aqueous CHG, and 10% aqueous PVI. However, the usual 2% CHG concentration was not assessed.^12^ The superior efficacy of high concentration may be attributed to dose-dependent microbicidal activity of CHG.^40,41,45,48,52,53^ CHG disrupts bacterial membranes, and higher concentrations ensure deeper penetration and prolonged bactericidal action.^54^

Temporal stability of effect estimates may also be associated with improvements in insertion and maintenance bundles over the years, which have reduced baseline infection rates in more recent trials. While these broader changes fall outside the scope of our analysis, they may partly explain the smaller effect sizes and narrower confidence intervals observed after 2010. One trial was published after 2020, limiting the ability to extrapolate findings for the most recent decade.^40^

SUCRA rankings summarize the probability that each intervention performs among the best but should be interpreted cautiously, as they do not reflect clinically meaningful differences. In particular, rankings involving aqueous CHG may be influenced by methodological factors and by the use, in some ICU trials, of a low-concentration CHG-benzalkonium chloride-benzyl alcohol solution that may artificially enhance its apparent efficacy.^55^ Because SUCRA is highly sensitive to imprecision and network structure, we used it for descriptive purposes only and based our conclusions primarily on relative treatment effects (RRs and absolute risk reductions), which was in line with current methodological guidance.^56,57,58^

IPA-based formulations were associated with lower risks of CRIs than ethanol-based solutions. In vitro, IPA is slightly more bactericidal than ethanol against *Escherichia coli* and *Staphylococcus aureus*.^59^ IPA has lower vapor pressure (approximately 33 mm Hg) than ethanol (approximately 44 mm Hg) at room temperature, implying slower evaporation and longer effective contact time.^60,61^ IPA is also more lipophilic than ethanol, a property that may enhance disruption of cutaneous lipids and reduction of resident skin flora.^14^

The interpretation of adverse events was limited by heterogeneous definitions and nonsystematic reporting. Criteria for cutaneous reactions varied from mild erythema to significant edema with vesicles, hampering comparisons between antiseptics. However, none of the reactions required specific treatment, and all disappeared once antiseptic application was stopped. Available data suggest CHG is as safe as PVI, with no clear increase in local reactions.^62^ Minor adverse events were likely driven by the alcohol component rather than by CHG or PVI themselves, as alcohol transiently increases skin permeability, removes surface lipids, and can induce irritation or erythema. This mechanism is consistent with the higher rate of cutaneous reactions observed with alcoholic formulations across several trials. CHG allergy, although reported, remains uncommon and is typically limited to mild contact dermatitis, with severe reactions being exceptional. Concerns regarding reduced susceptibility to CHG have also been raised in laboratory settings, but current clinical evidence does not demonstrate a meaningful loss of effectiveness, particularly for alcohol-based formulations.^62^

In this study, antiseptic strategy was not associated with hospital stay or mortality. These outcomes are multifactorial—driven by patient condition, infection severity, and management—making the direct effect of skin antisepsis in preventing CRIs difficult to isolate. The rarity of CRBSIs and unmeasured confounders such as comorbidities likely further reduced power to detect downstream effects. Still, because CRBSIs increase morbidity and prolong hospitalization, optimal antiseptic selection could improve outcome and reduce health care burden.^8,9,63,64^ Notably, no study reported time to infection onset, an outcome relevant for understanding antiseptic kinetics and guiding practice.

### Strengths and Limitations

The overall quality of evidence in this NMA is limited by clinical and methodological heterogeneity, reflecting variation in clinical practices across settings. Several factors may have contributed, including differences in antiseptic concentration and formulations; various catheter types with varying dwell times and infection risks; diverse patient populations (wards vs ICU patients); and nonstandardized application protocols (contact time, technique of application, drying time). In addition, low- and middle-income countries were underrepresented, limiting applicability to settings with different resources, epidemiology, and practices. These elements collectively explain observed variability. Despite these limitations, measures were taken to ensure reliability. Transitivity and coherence were assessed with no major violations; minor node-splitting inconsistencies, mainly where direct evidence was scarce, were mitigated by subgroup and sensitivity analyses. For catheter tip colonization, the global inconsistency test indicated disagreement between designs, likely reflecting heterogeneity in study protocols and antiseptic formulations. However, no significant local inconsistency was detected, and the primary conclusions of the network remained unchanged. Objective outcomes, such as CRBSIs and catheter tip colonization, further supported internal validity. Nevertheless, variation in sample sizes and methodological quality, particularly randomization and blinding, introduced risk of bias that should be considered when interpreting results. Although NMA integrates direct and indirect evidence across a broad network, it inevitably carries uncertainty where head-to-head trials are lacking.

The search strategy was developed in collaboration with an information specialist, delivering a high-quality and exhaustive search, minimizing missed studies and enhancing the reliability of the evidence base. To our knowledge, this review included the largest number of intravascular catheters analyzed on this topic. Inclusion of catheters of all types and patients of all ages and hospitalization sites, reflecting clinical practice, contributes to the robustness and clinical relevance of the findings. The rigorous methodology, including risk-of-bias assessment, sensitivity analyses, and exploratory analyses, enhances the robustness of the findings. Unlike previous reviews, this study differentiates antiseptic types by concentration and formulation, offering clinically relevant insights on the role of alcohol and CHG concentration in CRI prevention. Additionally, by focusing on CRBSIs rather than CLABSIs, this review specifically evaluates infections with confirmed catheter origin, providing a more accurate and clinically meaningful assessment of the impact of skin antisepsis at the time of insertion. Finally, to our knowledge, this is the first report to examine the relative effectiveness of IPA vs ethanol in clinical settings.

This study has limitations. Some trials had unclear or high risk of bias, particularly for protocol deviations, missing data, and outcome measurement, which could affect certainty. A further limitation was the unit of analysis—some studies reported outcomes per patient, others per device. In trials with multiple catheters per patient, we assumed independence between catheters. This may slightly overestimate precision, but is common in device-related meta-analyses. We also excluded within-class comparisons (eg, CHG vs CHG and PVI vs PVI) to focus on clinically relevant decisions between distinct antiseptics. Although this reduced network connectivity and limited assessment of global coherence, it improved interpretability and clinical applicability. Handling zero-event studies was another challenge, particularly for rare events. We used the conventional 0.5 correction, consistent with Cochrane guidance, recognizing that smaller or empirical corrections may reduce bias when events are rare.^65,66^ Given the limited number of such studies and absence of double-zero-event trials, alternative corrections were unlikely to change results meaningfully. The included studies did not consistently report time-to-infection data or early CRI incidence, preventing an analysis restricted to infections occurring within the first days after insertion, which would have been the most relevant timeframe for assessing the direct effect of skin antisepsis. Finally, as with any NMA, reliance on indirect comparisons introduces uncertainty where direct head-to-head evidence is lacking.

## Conclusions

These findings support most international guidelines favoring alcoholic formulations of high concentration alcoholic CHG over PVI for site disinfection before intravascular catheter insertion. They also suggest that IPA was superior to ethanol. However, evidence mainly comes from general and ICU populations, with scarce data in vulnerable groups, such as pediatric or immunocompromised patients. This gap limits generalizability, as efficacy, tolerance, and long-term safety might differ. Furthermore, economic and environmental aspects have rarely been considered in studies, which may be problematic for low- and middle-income countries. More studies are needed to confirm these recommendations, accounting for the efficacy, safety, cost, and sustainability of antiseptics.

## Notes

Supplement 1. **eAppendix 1.** Systematic Research Strategy **eAppendix 2.** List of Excluded Full-Text Articles With Reasons for Exclusion **eAppendix 3.** Detailed Data Analysis **eAppendix 4.** Assessment of Transitivity and Coherence in the Network Meta-Analysis **eAppendix 5.** Network Plots **eAppendix 6.** Surface Under the Cumulative Ranking Curve (SUCRA) Analysis **eAppendix 7.** Sensitivity Analyses **eAppendix 8.** Secondary Outcome Analysis and Subgroup Analysis **eAppendix 9.** Assessment of Risk of Bias and Reporting Bias

Supplement 2.
Data Sharing Statement
