# Supplementary material for: Chlorhexidine vs Povidone-Iodine and Incidence of Catheter-Related Infections: A Systematic Review and Meta-Analysis
Source: JAMA Netw Open. 2026 Feb 12;9(2):e2558954. doi: 10.1001/jamanetworkopen.2025.58954 (PMC12902894; doi:10.1001/jamanetworkopen.2025.58954)
Supplement: Supplement 1. — eAppendix 1. Systematic Research Strategy eAppendix 2. List of Excluded Full-Text Articles With Reasons for Exclusion eAppendix 3. Detailed Data Analysis eAppendix 4. Assessment of Transitivity and Coherence in the Network Meta-Analysis eAppendix 5. Network Plots eAppendix 6. Surface Under the Cumulative Ranking Curve (SUCRA) Analysis eAppendix 7. Sensitivity Analyses eAppendix 8. Secondary Outcome Analysis and Subgroup Analysis eAppendix 9. Assessment of Risk of Bias and Reporting Bias [file jamanetwopen-e2558954-s001.pdf]

Supplemental Online Content

Drugeon B, Mihala G, Schults J, et al. Chlorhexidine vs povidone-iodine and incidence of catheter-related infections: a systematic review and meta-analysis. *JAMA Netw Open*. 2026;9(2):e2558954. doi:10.1001/jamanetworkopen.2025.58954

eAppendix 1. Systematic Research Strategy

eAppendix 2. List of Excluded Full-Text Articles With Reasons for Exclusion

eAppendix 3. Detailed Data Analysis

eAppendix 4. Assessment of Transitivity and Coherence in the Network Meta-Analysis

eAppendix 5. Network Plots

eAppendix 6. Surface Under the Cumulative Ranking Curve (SUCRA) Analysis

eAppendix 7. Sensitivity Analyses

eAppendix 8. Secondary Outcome Analysis and Subgroup Analysis

eAppendix 9. Assessment of Risk of Bias and Reporting Bias

This supplemental material has been provided by the authors to give readers additional information about their work.

© 2026 Drugeon B et al. *JAMA Network Open*.

eAppendix 1. Systematic research strategy

Data search strategies were developed with an information specialist (NBa). To identify unpublished or ongoing trials, we also searched trial registries including ClinicalTrials.gov and the World Health Organization (WHO) International Clinical Trials Registry Platform. No date or language restrictions were applied. We did not undertake citation snowballing (backward reference checking or forward citation tracking).

Date searched: January 7, 2025

Total number of results = 1,073
Deduplicated number of results = using Covidence to remove duplicates

PubMed 182 results
Includes MeSH

("Chlorhexidine"[Mesh] OR “chlorhexidine”[tiab]) AND ("Povidone-Iodine"[Mesh] OR “PVP-I”[tiab] OR “PVPI”[tiab] OR “PVP-Iodine”[tiab] OR “PVP-Iodines”[tiab] OR “polyvinylpyrrolidone”[tiab] OR “povidone”[tiab] OR “povidones”[tiab] OR “iodine”[tiab] OR “iodines”[tiab] OR “povidone-iodine”[tiab] OR “povidone-iodines”[tiab] OR “iodopovidone”[tiab] OR “iodopovidones”[tiab]) AND ("Catheters"[Mesh] OR "Catheterization"[Mesh] OR "Catheterization, Central Venous"[Mesh] OR "Catheterization, Peripheral"[Mesh] OR "Catheter-Related Infections"[Mesh] OR “catheter”[tiab] OR “catheters”[tiab] OR “cannula”[tiab] OR “cannulas”[tiab] OR “cannulae”[tiab] OR “catheterisation”[tiab] OR “catheterisations”[tiab] OR “catheterization”[tiab] OR “catheterizations”[tiab] OR “cannulation”[tiab] OR “cannulations”[tiab] OR “vascular access”[tiab] OR “venous access”[tiab] OR “central line”[tiab] OR “central lines”[tiab] OR “midline”[tiab] OR “midlines”[tiab] OR “intravascular”[tiab] OR “intravenous”[tiab] OR “venous line”[tiab] OR “venous lines”[tiab] OR “PVC”[tiab] OR “PVCs”[tiab] OR “PVAC”[tiab] OR “PVACs”[tiab] OR “PIC”[tiab] OR “PICs”[tiab] OR “PIVC”[tiab] OR “PIVCs”[tiab] OR “VAD”[tiab] OR “VADS”[tiab] OR “PICC”[tiab] OR “PICCs”[tiab] OR “CVAD”[tiab] OR “CVADs”[tiab] OR “CVC”[tiab] OR “CVCs”[tiab]) NOT (animals [mh] NOT humans [mh])

CINAHL Complete (EBSCOhost) 108 results
Includes CINAHL Subject Headings

(MH "Chlorhexidine" OR TI(“chlorhexidine”) OR AB(“chlorhexidine”)) AND (MH "Povidone-Iodine" OR TI(“PVP-I” OR “PVPI” OR “PVP-Iodine” OR “PVP-Iodines” OR “polyvinylpyrrolidone” OR “povidone” OR “povidones” OR “iodine” OR “iodines” OR “povidone-iodine” OR “povidone-iodines” OR “iodopovidone” OR “iodopovidones”) OR AB(“PVP-I” OR “PVPI” OR “PVP-Iodine” OR “PVP-Iodines” OR “polyvinylpyrrolidone” OR “povidone” OR “povidones” OR “iodine” OR “iodines” OR “povidone-iodine” OR “povidone-iodines” OR “iodopovidone” OR “iodopovidones”)) AND (MH "Catheters+" OR MH "Catheterization+" OR MH "Catheter-Related Infections+" OR TI(“catheter” OR “catheters” OR “cannula” OR “cannulas” OR “cannulae” OR “catheterisation” OR “catheterisations” OR “catheterization” OR “catheterizations” OR “cannulation” OR “cannulations” OR “vascular access” OR “venous access” OR “central line” OR “central lines” OR “midline” OR “midlines” OR “intravascular” OR “intravenous” OR “venous line” OR “venous lines” OR “PVC” OR “PVCs” OR “PVAC” OR “PVACs” OR “PIC” OR “PICs” OR “PIVC” OR “PIVCs” OR “VAD” OR “VADS” OR “PICC” OR “PICCs” OR “CVAD” OR “CVADs” OR “CVC” OR “CVCs”) OR AB(“catheter” OR “catheters” OR “cannula” OR “cannulas” OR “cannulae” OR “catheterisation” OR “catheterisations” OR “catheterization” OR “catheterizations” OR “cannulation” OR “cannulations” OR “vascular access” OR “venous access” OR “central line” OR “central lines” OR “midline” OR “midlines” OR “intravascular” OR “intravenous” OR “venous line” OR “venous lines” OR “PVC” OR “PVCs” OR “PVAC” OR “PVACs” OR “PIC” OR “PICs” OR “PIVC” OR “PIVCs” OR “VAD” OR “VADS” OR “PICC” OR “PICCs” OR “CVAD” OR “CVADs” OR “CVC” OR “CVCs”)) NOT ((MH "Animals+" OR MH "Animal Studies" OR TI animal model*) NOT MH "Human")

Embase (Elsevier) 278 results
Includes Emtree
Limited to relevant publication types (including articles and articles in press)

('chlorhexidine'/exp OR “chlorhexidine”:ti,ab) AND ('povidone iodine'/exp OR “PVP-I”:ti,ab OR “PVPI”:ti,ab OR “PVP-Iodine”:ti,ab OR “PVP-Iodines”:ti,ab OR “polyvinylpyrrolidone”:ti,ab OR “povidone”:ti,ab OR “povidones”:ti,ab OR “iodine”:ti,ab OR “iodines”:ti,ab OR “povidone-iodine”:ti,ab OR “povidone-iodines”:ti,ab OR “iodopovidone”:ti,ab OR “iodopovidones”:ti,ab) AND ('catheter'/exp OR 'catheterization'/exp OR 'blood vessel catheterization'/exp OR 'catheter infection'/exp OR “catheter”:ti,ab OR “catheters”:ti,ab OR “cannula”:ti,ab OR “cannulas”:ti,ab OR “cannulae”:ti,ab OR “catheterisation”:ti,ab OR “catheterisations”:ti,ab OR “catheterization”:ti,ab OR “catheterizations”:ti,ab OR “cannulation”:ti,ab OR “cannulations”:ti,ab OR “vascular access”:ti,ab OR “venous access”:ti,ab OR “central line”:ti,ab OR “central lines”:ti,ab OR “midline”:ti,ab OR “midlines”:ti,ab OR

© 2026 Drugeon B et al. *JAMA Network Open*.

"intravascular":ti,ab OR "intravenous":ti,ab OR "venous line":ti,ab OR "venous lines":ti,ab OR "PVC":ti,ab OR "PVCs":ti,ab OR "PVAC":ti,ab OR "PVACs":ti,ab OR "PIC":ti,ab OR "PICs":ti,ab OR "PIVC":ti,ab OR "PIVCs":ti,ab OR "VAD":ti,ab OR "VADS":ti,ab OR "PICC":ti,ab OR "PICCs":ti,ab OR "CVAD":ti,ab OR "CVADs":ti,ab OR "CVC":ti,ab OR "CVCs":ti,ab) AND ([article]/lim OR [article in press]/lim) NOT ('animal experiment'/de NOT ('human experiment'/de OR 'human'/de))

Cochrane Library (Wiley) 149 results in CENTRAL Trials
Includes MeSH
Advanced search > Search manager
Link to saved search - https://www.cochranelibrary.com/advanced-search/search-manager?search=7616160

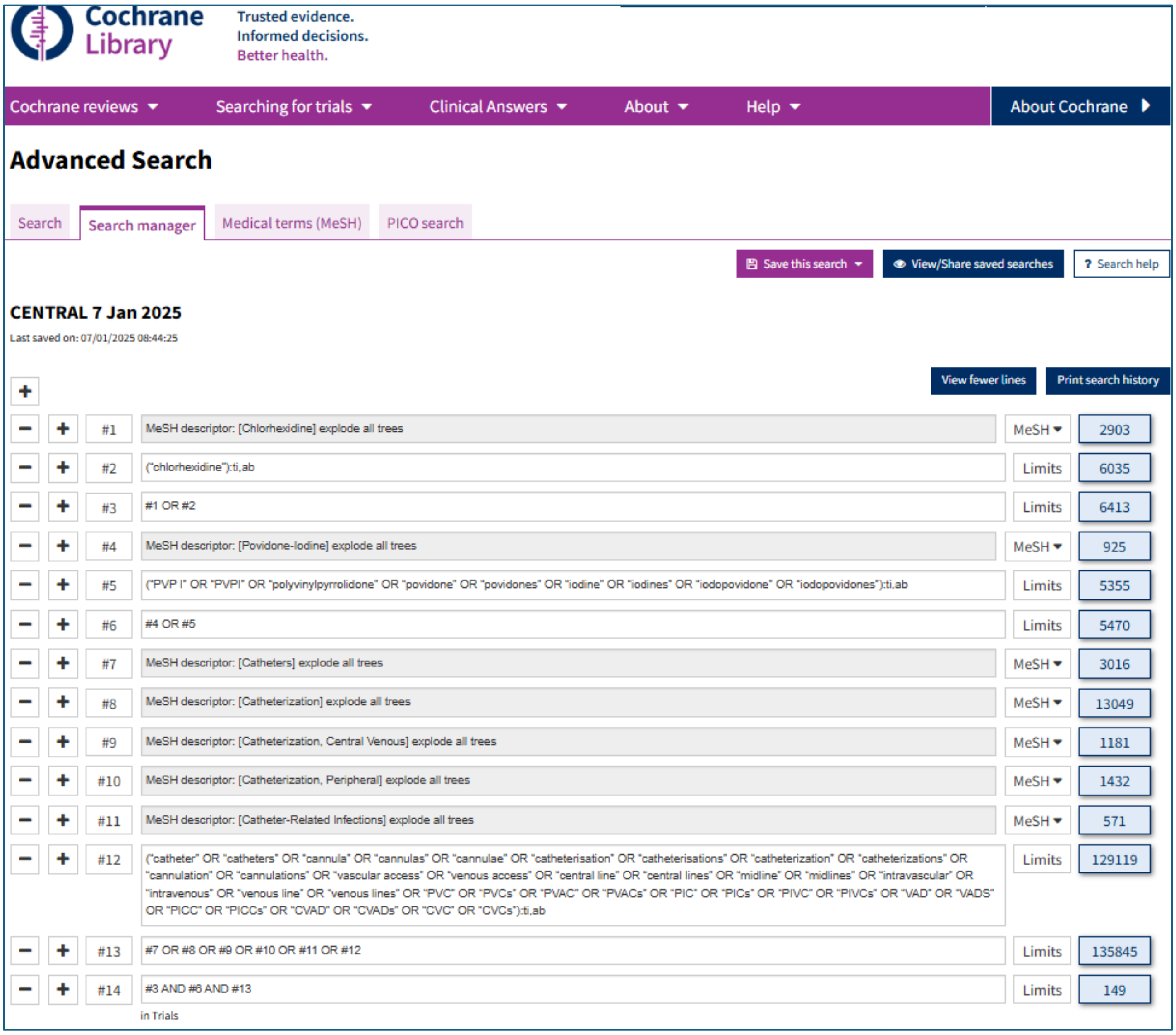

ID Search Hits
#1 MeSH descriptor: [Chlorhexidine] explode all trees 2903
#2 ("chlorhexidine"):ti,ab 6035
#3 #1 OR #2 6413
#4 MeSH descriptor: [Povidone-Iodine] explode all trees 925
#5 ("PVP I" OR "PVPI" OR "polyvinylpyrrolidone" OR "povidone" OR "povidones" OR "iodine" OR "iodines" OR "iodopovidone" OR "iodopovidones"):ti,ab 5355
#6 #4 OR #5 5470
#7 MeSH descriptor: [Catheters] explode all trees 3016
#8 MeSH descriptor: [Catheterization] explode all trees 13049
#9 MeSH descriptor: [Catheterization, Central Venous] explode all trees 1181
#10 MeSH descriptor: [Catheterization, Peripheral] explode all trees 1432

© 2026 Drugeon B et al. *JAMA Network Open*.

#11 MeSH descriptor: [Catheter-Related Infections] explode all trees 571
#12 (“catheter” OR “catheters” OR “cannula” OR “cannulas” OR “cannulae” OR “catheterisation” OR “catheterisations” OR “catheterization” OR “catheterizations” OR “cannulation” OR “cannulations” OR “vascular access” OR “venous access” OR “central line” OR “central lines” OR “midline” OR “midlines” OR “intravascular” OR “intravenous” OR “venous line” OR “venous lines” OR “PVC” OR “PVCs” OR “PVAC” OR “PVACs” OR “PIC” OR “PICs” OR “PIVC” OR “PIVCs” OR “VAD” OR “VADS” OR “PICC” OR “PICCs” OR “CVAD” OR “CVADs” OR “CVC” OR “CVCs”):ti,ab 129119
#13 #7 OR #8 OR #9 OR #10 OR #11 OR #12 135845
#14 #3 AND #6 AND #13 in Trials 149

Scopus (Elsevier) 193 results
Advanced search

TITLE-ABS((“chlorhexidine”) AND (“PVP-I” OR “PVPI” OR “PVP-Iodine” OR “PVP-Iodines” OR “polyvinylpyrrolidone” OR “povidone” OR “povidones” OR “iodine” OR “iodines” OR “povidone-iodine” OR “povidone-iodines” OR “iodopovidone” OR “iodopovidones”) AND (“catheter” OR “catheters” OR “cannula” OR “cannulas” OR “cannulae” OR “catheterisation” OR “catheterisations” OR “catheterization” OR “catheterizations” OR “cannulation” OR “cannulations” OR “vascular access” OR “venous access” OR “central line” OR “central lines” OR “midline” OR “midlines” OR “intravascular” OR “intravenous” OR “venous line” OR “venous lines” OR “PVC” OR “PVCs” OR “PVAC” OR “PVACs” OR “PIC” OR “PICs” OR “PIVC” OR “PIVCs” OR “VAD” OR “VADS” OR “PICC” OR “PICCs” OR “CVAD” OR “CVADs” OR “CVC” OR “CVCs”))

Web of Science (Clarivate) 163 results
Web of Science Core Collection, All Editions
Advanced search > Query preview

(TI=(“chlorhexidine”) OR AB=(“chlorhexidine”)) AND (TI= (“PVP-I” OR “PVPI” OR “PVP-Iodine” OR “PVP-Iodines” OR “polyvinylpyrrolidone” OR “povidone” OR “povidones” OR “iodine” OR “iodines” OR “povidone-iodine” OR “povidone-iodines” OR “iodopovidone” OR “iodopovidones”) OR AB=(“PVP-I” OR “PVPI” OR “PVP-Iodine” OR “PVP-Iodines” OR “polyvinylpyrrolidone” OR “povidone” OR “povidones” OR “iodine” OR “iodines” OR “povidone-iodine” OR “povidone-iodines” OR “iodopovidone” OR “iodopovidones”)) AND (TI=(“catheter” OR “catheters” OR “cannula” OR “cannulas” OR “cannulae” OR “catheterisation” OR “catheterisations” OR “catheterization” OR “catheterizations” OR “cannulation” OR “cannulations” OR “vascular access” OR “venous access” OR “central line” OR “central lines” OR “midline” OR “midlines” OR “intravascular” OR “intravenous” OR “venous line” OR “venous lines” OR “PVC” OR “PVCs” OR “PVAC” OR “PVACs” OR “PIC” OR “PICs” OR “PIVC” OR “PIVCs” OR “VAD” OR “VADS” OR “PICC” OR “PICCs” OR “CVAD” OR “CVADs” OR “CVC” OR “CVCs”) OR AB=(“catheter” OR “catheters” OR “cannula” OR “cannulas” OR “cannulae” OR “catheterisation” OR “catheterisations” OR “catheterization” OR “catheterizations” OR “cannulation” OR “cannulations” OR “vascular access” OR “venous access” OR “central line” OR “central lines” OR “midline” OR “midlines” OR “intravascular” OR “intravenous” OR “venous line” OR “venous lines” OR “PVC” OR “PVCs” OR “PVAC” OR “PVACs” OR “PIC” OR “PICs” OR “PIVC” OR “PIVCs” OR “VAD” OR “VADS” OR “PICC” OR “PICCs” OR “CVAD” OR “CVADs” OR “CVC” OR “CVCs”))

© 2026 Drugeon B et al. *JAMA Network Open*.

eAppendix 2. List of excluded full-text articles with reasons for exclusion.

References	Reason for exclusion
Bilir, A.; Yelken, B.; Erkan, A. Chlorhexidine, octenidine or povidone iodine for catheterrelated infections: a randomised controlled trial. Critical care.2009;13:S79. DOI: 10.1186/cc7358.	Abstract Conference
Bilir, A.; Yelken, B.; Erkan, A. Cholorhexidine, octenidine or povidone iodine for catheterrelated infections: a randomised controlled trial. Journal of Research in Medical Sciences 2013;18(6):510-512	Wrong outcomes
Chaiyakunapruk, N.; Veenstra, D. L.; Lipsky, B. A.; Saint, S.; Chaiyakunapruk, Nathorn; Veenstra, David L.; Lipsky, Benjamin A.; Saint, Sanjay. Chlorhexidine compared with povidone-iodine solution for vascular catheter-site care: a meta-analysis. Annals of Internal Medicine 2002;136(11):792-I26. DOI: 10.7326/0003-4819-136-11-200206040-00007	Review article
Chaiyakunapruk, N.; Veenstra, D. L.; Lipsky, B. A.; Sullivan, S. D.; Saint, S. The clinical and economic benefits of chlorhexidine compared to povidone-iodine for vascular catheter site care JOURNAL OF GENERAL INTERNAL MEDICINE APR 2002;17():136-136	Wrong study design
Cheyron, Damien; Parienti, Jean-Jacques; Pages, Justine; Dutheil, Jean-Jacques; Fournel, François; Hazera, Pascal; Mégarbane, Bruno; Thuong, Marie; Valette, Xavier; Daubin, Cédric; Mermel, Leonard; Mira, Jean-Paul; Mégarbane, Bruno; du Cheyron, Damien; Fournel, François; Mermel, Leonard A.; Daubin, Cédric. Comparison of alcoholic chlorhexidine and povidone-iodine cutaneous antiseptics for the prevention of central venous catheter-related infection: a cohort and quasi-experimental multicenter study. Intensive Care Medicine 2016;42(9):1418-1426. DOI: 10.1007/s00134-016-4406-4	Wrong study design
Cobett, S.; LeBlanc, A. IV site infection: a prospective, randomized clinical trial comparing the efficacy of three methods of skin antisepsis: CINA conference '99. CINA: Official Journal of the Canadian Intravenous Nurses Association 1999;15():48-49	Abstract conference (full article available and selected)
A study to compare the efficacy of antiseptics Chlorhexidine alcohol vs Povidone Iodine alcohol in reducing bacterial colonisation by intravenous catheter in Paediatric ward in hospital. Ctri, https://trialsearch.who.int/Trial2.aspx?TrialID=CTRI/2023/03/050292 2023;():	Protocol
Dickenson, L. Central venous catheter site care: chlorhexidine vs. povidone-iodine. ANNA journal.1997;24(3):349, 358	Wrong study design
Euctr, G. B.. Open label Randomised Controlled Trial comparing povidone-iodine 10% with alcohol and 0.5% chlorhexidine with alcohol for prevention of early infection associated with insertion of central venous access devices - RCT for the prevention of early CVAD Infection https://trialsearch.who.int/Trial2.aspx?TrialID=EUCTR2006-000675-15-GB 2006;():	Protocol
Euctr, I. E. To compare the ability of two antiseptic agents (Chlorohexidine and Povidone-iodine), when applied to the skin prior to insertion of a tube into a premature infant, to prevent infections in the blood stream https://trialsearch.who.int/Trial2.aspx?TrialID=EUCTR2011-002962-19-IE 2011;():	Protocol
Garland, J. S.; Buck, R. K.; Maloney, P.; Durkin, D. M.; Toth-Lloyd, S.; Duffy, M.; Szocik, P.; McAuliffe, T. L.; Goldmann, D. Comparison of 10% povidone-iodine and 0.5% chlorhexidine gluconate for the prevention of peripheral intravenous catheter colonization in neonates: A prospective trial. Pediatric Infectious Disease Journal 1995;14(6):510-516	Wrong study design
Garland, J. S.; Buck, R. K.; Maloney, P.; Goldmann, D. A. Prospective trial of povidone-iodine (pi) versus chlorhexidine gluconate (c) for prevention of local peripheral intravenous catheter infections (lci) in neonates. Pediatric research. 1994;35(4):A226-A226	Wrong study design
Girard, R.; Comby, C.; Jacques, D. Alcoholic povidone-iodine or chlorhexidine-based antiseptic for the prevention of central venous catheter-related infections: In-use comparison. Journal of Infection and Public Health 2012;5(1):35-42. DOI: 10.1016/j.jiph.2011.10.007	Wrong study design
Goudet, Véronique; Timsit, Jean-François; Lucet, Jean-Christophe; Lepape, Alain; Balayn, Dorothée; Seguin, Sabrina; Mimoz, Olivier. Comparison of four skin preparation strategies to prevent catheter-related infection in intensive care unit (CLEAN trial): a study protocol for a randomized controlled trial. Trials 2013;14(1):114-114. DOI: 10.1186/1745-6215-14-114	Protocol
The comparison of chlorhexidine and povidone-iodine alcohol on bacterial colonization after peripheral vascular catheter insertion. Irct2017062034653N, https://trialsearch.who.int/Trial2.aspx?TrialID=IRCT2017062034653N1 2017;():	Protocol
Randomised trial comparing 10% povidone-iodine with alcohol and 0.5% chlorhexidine with 70% alcohol for prevention of early infection associated with central venous catheter insertion Isrctn, https://trialsearch.who.int/Trial2.aspx?TrialID=ISRCTN51113796 2006;():	Protocol
Jeffries, I. P.; Salas, A.; Chandler, B.; Soliz, A. SHORT TERM outcomes with use of chlorhexidine gluconate (chg) and povidone-iodine (pi) in vlbwi with percutaneously placed central venous catheters. Pediatric research. 2010;68():241-241.DOI: 10.1203/00006450-201011001-00471	Wrong study design
Kao, Hsiang-Fong; Chen, I. Chun; Hsu, Chiun; Chang, Sin-Yuan; Chien, Shu-Fen; Chen, Yee-Chun; Hu, Fu-Chang; Yang, James Chih-Hsin; Cheng, Ann-Lii; Yeh, Kun-Huei. Chlorhexidine for the prevention of bloodstream infection associated with totally implantable venous ports in patients with solid cancers Supportive Care in Cancer. 2014;22(5):1189-1197. DOI: 10.1007/s00520-013-2071-5	Wrong study design
Kieran, E.; Miletin, J.; Twomey, A.; Knowles, S.; O' Donnell, C. Randomized trial of chlorhexidine versus povidone-iodine for skin antisepsis prior to central venous catheter insertion in preterm infants (Eudract 2011-002962-19) Pediatric academic societies (PAS) annual meeting; 2015 apr 25 - 28; san diego, USA 2015;():	Abstract conference (full article available and selected)
Kulkarni, A. P.; Awode, R. M. A prospective randomised trial to compare the efficacy of povidone-iodine 10% and chlorhexidine 2% for skin disinfection. Indian Journal of Anaesthesia. 2013;57(3):270-275. DOI: 10.4103/0019-5049.115619	Wrong outcomes
Lai, N. M.; Lai, N. A.; O'Riordan, E.; Chaiyakunapruk, N.; Taylor, J. E.; Tan, K. Skin antisepsis for reducing central venous catheter-related infections. Cochrane Database of Systematic Reviews 2016;2016(7). DOI: 10.1002/14651858.CD010140.pub2	Review article
LeBlanc, A.; Cobbett, S. Traditional practice versus evidence-based practice for IV skin preparation. Canadian Journal of Infection Control / Revue Canadienne de Prévention des Infections.2000;15(1):9-14	Wrong study design

© 2026 Drugeon B et al. *JAMA Network Open*.

Lin, M. R.; Chang, P. J.; Hsu, P. C.; Lin, C. S.; Chiu, C. H.; Chen, C. J. Comparison of Efficacy of 2% Chlorhexidine Gluconate–Alcohol and 10% Povidone-Iodine–Alcohol against Catheter-Related Bloodstream Infections and Bacterial Colonization at Central Venous Catheter Insertion Sites: a Prospective, Single-Center, Open-Label, Crossover Study. Journal of Clinical Medicine 2022;11(8). DOI: 10.3390/jcm11082242	Wrong study design
Maki, D. G.; Ringer, M.; Alvarado, C. J. Prospective randomized trial of povidone-iodine, alcohol, and chlorhexidine for prevention of infection associated with central venous and arterial catheters<br>AJIC. 1994;22(4):242. DOI: 10.1016/0196-6553(94)90073-6	Commentary on published article
Maki, D. G.; Ringer, M.; Alvarado, C. J. Prospective randomized trial of povidone-iodine, alcohol, and chlorhexidine for prevention of infection associated with central venous and arterial catheters. CINA: Official Journal of the Canadian Intravenous Nurses Association 1993;9(1):10-15	Review article
Masuyama, T.; Yasuda, H.; Sanui, M.; Lefor, A. K. Systematic Review Effect of skin antiseptic solutions on the incidence of catheter-related bloodstream infection: a systematic review and network meta-analysis. Journal of Hospital Infection. 2021;110():156-164. DOI: 10.1016/j.jhin.2021.01.017	Review article
Maunoury, F.; Farinetto, C.; Ruckly, S.; Guenezan, J.; Lucet, J. C.; Lepape, A.; Pascal, J.; Souweine, B.; Mimoz, O.; Timsit, J. F. Cost-effectiveness analysis of chlorhexidine-alcohol versus povidone iodine-alcohol solution in the prevention of intravascular-catheter-related bloodstream infections in France. PLoS One 2018;13(5):e0197747. DOI: 10.1371/journal.pone.0197747	Wrong study design
Mimoz, O.; Lucet, J. C.; Kerforne, T.; Pascal, J.; Souweine, B.; Goudet, V.; Mercat, A.; Bouadma, L.; Lasocki, S.; Alfandari, S.; et al. Chlorhexidine-alcohol versus povidone iodine-alcohol antisepsis for catheter-related infection prevention: an open-label, multicentre, randomised controlled trial. Intensive care medicine experimental 2015;3. DOI: 10.1186/2197-425X-3-S1-A409	Abstract conference (full article available and selected)
Muhd Helmi, M. A.; Lai, N. M.; Van Rostenberghe, H.; Ayub, I.; Mading, E. Antiseptic solutions for skin preparation during central catheter insertion in neonates. Cochrane Database of Systematic Reviews 2023;2023(5). DOI: 10.1002/14651858.CD013841.pub2	Review article
Myaneh, Z. T.; Alizadeh, S. A.; Shahrokhi, A.; Rashvand, F. Comparing the Effects of Chlorhexidine 2% and Iodopovidone-alcohol on Peripheral Venous Catheter Bacterial Colonization in Preterm Neonates. Iranian Journal of Neonatology 2019;10(3):64-69. DOI: 10.22038/ijn.2019.35152.1531	Wrong intervention
Natsuo, Yamamoto; Hideo, Kimura; Hanako, Misao; Hayato, Matsumoto; Yuji, Imafuku; Akemi, Watanabe; Hiroko, Mori; Akiko, Yoshida; Saori, Miura; Yoshinobu, Abe; Mamoru, Toba; Hiromi, Suzuki; Kazuei, Ogawa; Keiji, Kanemitsu. Efficacy of 1.0% chlorhexidine-gluconate ethanol compared with 10% povidone-iodine for long-term central venous catheter care in hematology departments: A prospective study. AJIC: American Journal of Infection Control 2014;42(5):574-576. DOI: 10.1016/j.ajic.2013.12.023	Wrong outcomes
Prospective Randomized Controlled Multicenter Trial of 4 Antiseptic Strategies for Prevention of Catheter Infection in Intensive Care Unit for Adults Patients<br>Nct,<br>https://clinicaltrials.gov/show/NCT01629550 2012;():	Protocol
Chlorhexidine-Alcohol Versus Povidone Iodine-Alcohol, Combined or Not With Use of a Bundle of New Devices, for Prevention of Intravascular-catheter Colonization and Catheter Failure<br>Nct,<br>https://clinicaltrials.gov/show/NCT03757143 2018;():	Protocol
Paglialonga, F.; Consolo, S.; Biasuzzi, A.; Assomou, J.; Gattarello, E.; Patricelli, M. G.; Giannini, A.; Chidini, G.; Napolitano, L.; Edefonti, A.<br>Reduction in catheter-related infections after switching from povidone-iodine to chlorhexidine for the exit-site care of tunneled central venous catheters in children on hemodialysis. Hemodial Int Oct 2014;18 Suppl 1():S13-8. DOI: 10.1111/hdi.12218	Wrong study design
Perier, A.; Kriegel, I.; Botrel, B.; Borne, M.; Queinnec, M.; Marcou, A.; Goater, P.; Kirov, K.; Ghimouz, M.; Guillaume, A.; Esteve, M. Precocious infections in implantable catheters (ICC): comparison of cutaneous preparation by 2% chlorhexidine and 70% isopropanol (CHX-OH) in one stage or iodized povidone alcohol. ANNALES FRANCAISES D ANESTHESIE ET DE REANIMATION SEP 2013;32():A326-A326. DOI: 10.1016/j.annfar.2013.07.608	Wrong study design
Ridling, D. A.; Zimmerman, J.; Kathy, G. PICU nosocomial line infections (NLI) using 10% povidone-iodine (PI) and 70% alcohol (AL) vs 2% chlorhexidine (CHG), for central line placement and maintenance. Critical Care Medicine DEC 2000;28(12):A84-A84	Full text unavailable
Roebuck, A. A 0.5% chlorhexidine gluconate in 70% isopropyl alcohol swab was more effective than 2 other methods for intravenous skin antisepsis...commentary on LeBlanc A, Cobbett S. Traditional practice versus evidence-based practice for IV skin preparation. CAN J INFECT. 2000;():119-119	Commentary on published article
Schrover, I. M.; Spronk, P. E. Chlorhexidine is better than iodine as disinfectant for central venous catheter care. Nederlands Tijdschrift voor Geneeskunde 2008;152(9):527	Commentary on published article
Shi, Yu; Yang, Ning; Zhang, Li; Zhang, Ming; Pei, Hong-Hong; Wang, Hai. Chlorhexidine disinfectant can reduce the risk of central venous catheter infection compared with povidone: a meta-analysis. AJIC: American Journal of Infection Control 2019;47(10):1255-1262. DOI: 10.1016/j.ajic.2019.02.024	Review article
Smotrich, L. A Comparison of Chlorhexidine Gluconate Versus Povidone-Iodine for Umbilical Catheter Placement in the Extremely Low Birth-Weight Infant. Advances in Neonatal Care JUN 2017;17(3):E19-E20. DOI: 10.1097/ANC.0000000000000407	Wrong study design
Timsit, J. F.; L'Hériteau, F.; Lepape, A.; Francais, A.; Ruckly, S.; Venier, A. G.; Jarno, P.; Boussat, S.; Coignard, B.; Savey, A.; Timsit, J. F.; L'Hériteau, F.; Lepape, A.; Francais, A.; Ruckly, S.; Venier, A. G.; Jarno, P.; Boussat, S.; Coignard, B.; Savey, A. A multicentre analysis of catheter-related infection based on a hierarchical model. Intensive Care Medicine 2012;38(10):1662-1672. DOI: 10.1007/s00134-012-2645-6	Wrong study design
Van Esch, J. Chlorhexidine reduced catheter tip colonisation more than 10% povidone-iodine in critically ill neonates<br>Evidence Based Nursing 2002;5(3):73-73	Wrong intervention
Yasuda, H.; Sanui, M.; Komuro, T.; Hatakeyama, J.; Matsukubo, S.; Kawano, S.; Yamamoto, H.; Andoh, K.; Seo, R.; Shime, N.; et al. Comparison of three cutaneous antiseptic solutions for the prevention of catheter colonization in an ICU for adult patients: a multicenter prospective randomized controlled trial. Critical care. 2015;19():S26. DOI: 10.1186/cc14153	Abstract conference (full article available and selected)

© 2026 Drugeon B et al. *JAMA Network Open*.

Yasuda, H.; Sanui, M.; Komuro, T.; Kawano, S.; Andoh, K.; Yamamoto, H.; Noda, E.; Hatakeyama, J.; Saitou, N.; Okamoto, H.; et al. Comparison of the efficacy of three cutaneous antiseptic solutions for preventing catheter colonization: a multicenter, prospective, open-label, parallel, randomized controlled study. Intensive care medicine experimental 2016;4. DOI: 10.1186/s40635-016-0100-7	Abstract conference (full article available and selected)
Yentür, A.; Topçu, I.; Işik, R.; Değerli, K.; Sürücüoğlu, S. Underestimated role of alcohol at skin disinfection: Lipid dissolving property when used in association with conventional antiseptic agents. Turkish Journal of Medical Sciences 2010;40(4):593-598. DOI: 10.3906/sag-0904-50	Wrong intervention
Zhang, F.; Xiao, Y.; Wang, Q. Effects of two disinfectants on preventing central line associated blood stream infection. Blood Purification 2018;45(4):38. DOI: 10.1159/000486831	Wrong study design

© 2026 Drugeon B et al. *JAMA Network Open*.

eAppendix 3. Detailed data analysis.

Data were extracted independently by two reviewers from the same team of four individuals, using a standardized data extraction form in Covidence®. In the event of discrepancies between the two, a third reviewer from the same team independently re-extracted the data to resolve disagreement. Data were collated in Microsoft Excel® (Microsoft, Washington) for statistical analysis.

Continuous variables were presented as mean and standard deviation (SD) or median and interquartile range (IQR). Categorical variables were described as numbers and percentages. Tables were used to summarize study characteristics.

For continuous outcomes, when studies reported medians and IQRs instead of means and SDs, we estimated the corresponding mean and SD using established methods based on the reported sample size and distribution quantiles (1–3). Attempts were made to contact the corresponding authors for clarification or unpublished data.

In studies comparing more than two antiseptic strategies, the comparator group was split proportionally (and rounded down to the nearest whole number if necessary) to avoid overrepresentation in the analysis.

In studies where one treatment arm reported zero events for a given outcome, a continuity correction of 0.5 was applied to enable calculation of effect estimates and to avoid introducing bias through exclusion. Studies with double-zero-event arms were excluded from the quantitative synthesis (4,5). All eligible studies were included in the review. For each outcome, studies were analyzed whenever quantitative synthesis was feasible; if only a single study reported the outcome, or if meta-analysis was not possible, the findings were presented narratively. Analyses were performed using R software (version 4.2.1 [2022-06-23], Biocore, Boston) with the package *Metafor* (6), *Igraph* (7), and *netmeta* (8). A p-value < 0.05 was considered statistically significant.

NMAs were conducted to allow for the simultaneous analysis of direct and indirect comparisons, providing a comprehensive assessment of the relative effectiveness of the included interventions. For the NMA, we included all randomized trials comparing CHG-based with PVI-based skin antisepsis. Each treatment arm was classified into a predefined node according to antiseptic agent (CHG vs PVI), CHG concentration (high [≥1%] vs low [<1%]), formulation (alcohol-based vs aqueous), and type of alcohol (isopropyl vs ethanol). Each unique combination of these characteristics constituted a distinct intervention node. Head-to-head CHG–PVI comparisons from individual trials defined the edges between nodes. Although the protocol prespecified a multilevel meta-analysis with meta-regression as an alternative strategy if the evidence base proved insufficient to construct a coherent network, this approach was not required. After study selection, the network demonstrated adequate connectivity and methodological assumptions were met, supporting the use of NMA as the primary analytical framework.

Given the anticipated clinical and methodological heterogeneity across studies, particularly in terms of patient populations, catheter types, antiseptic protocols, and clinical settings, a random-effects model using restricted maximum likelihood (REML) estimation was applied within a frequentist framework for all NMA. Effect estimates for each pairwise comparison were expressed as relative risks (RRs) with 95% confidence intervals (CIs) for dichotomous outcomes, and mean differences (MDs) for continuous outcomes.

Transitivity was assessed by examining whether the distribution of key effect modifiers was comparable across the different CHG–PVI treatment comparisons. The main potential modifiers identified a priori were catheter type (peripheral *vs.* central), clinical setting (ICU vs non-ICU), CHG concentration (high *vs.* low), and antiseptic formulation (alcohol-based *vs*. aqueous). These variables showed broadly similar distributions across comparison groups, supporting the plausibility of the transitivity assumption for the primary outcomes. Differences in catheter dwell time were expected due to inherent clinical differences between catheter types and were not judged to threaten transitivity.

© 2026 Drugeon B et al. *JAMA Network Open*.

Local inconsistency was assessed through node-splitting, comparing direct and indirect estimates for each comparison. Global inconsistency was evaluated using the design-by-treatment interaction model, which tests for disagreement across the entire network.

Statistical heterogeneity was assessed using the I^2 statistic and its 95% CI, with values >75% considered indicative of substantial heterogeneity (9,10). In addition, between-study variance was quantified using the REML-estimated τ^2 and its 95% CI. A τ^2 value >0.5 was interpreted as reflecting high dispersion of true effects across studies. For the primary outcome, a 95% prediction interval (PI) was calculated to reflect the expected range of true effects in future comparable settings.

A network plot was generated for each outcome to visually represent the distribution of comparisons.

To rank the different antiseptic strategies based on their efficacy, we performed a Surface Under the Cumulative Ranking Curve (SUCRA) analysis within the NMA framework. SUCRA values range from 0 to 100%, with higher values reflecting greater probability of an antiseptic being the most effective option. This analysis was applied to the primary outcome (catheter-related infections, including CRBSI, catheter tip colonization, and local infections) to establish a comparative ranking of antiseptics. In addition, a rankogram was constructed to visually represent the probability distribution of each antiseptic strategy across possible ranks, highlighting the degree of uncertainty around their estimated ranking.

To assess the robustness of findings and explore potential sources of heterogeneity, we conducted a series of subgroup, sensitivity, and exploratory analyses, all focusing on CRI outcomes.

Subgroup analyses were pre-specified and conducted within the NMA framework. These analyses stratified studies based on key clinical characteristics, including patient population (adults vs. infants), and catheter type (peripheral intravenous catheters [PIVCs] vs. central catheters, including CVCs, DCs, PICCs, ACs, and PACs). This approach allowed for an assessment of whether the relative efficacy of antiseptic formulations varied by population or device type.

Sensitivity analyses were performed post hoc to evaluate the stability of the NMA findings. First, we excluded studies deemed at high risk of bias according to the RoB2 tool (e.g., issues with randomization, protocol deviations, or missing data). The analyses were then repeated to determine whether the exclusion of these studies altered the overall results. Second, we conducted a conventional pairwise meta-analysis comparing CHG versus PVI, regardless of concentration or formulation, using a random-effects model. This analysis provided a complementary perspective based solely on direct evidence, independent of the transitivity and consistency assumptions inherent to the NMA framework.

Exploratory analyses were also conducted within the NMA to investigate formulation- and concentration-specific effects. First, we performed a network-based subgroup analysis comparing high-concentration versus low-concentration CHG formulations to assess potential differences in efficacy. This exploratory analyze aimed to provide mechanistic insights and to assess whether antiseptic concentration or formulation characteristics modified treatment effects. Second, we compared alcohol-based versus aqueous-based antiseptics within the NMA, regardless of the active compound, to isolate the impact of alcohol as a formulation component. Third, we compared ethanol- versus isopropyl alcohol–based formulations (EtOH vs IPA), irrespective of the active antiseptic, to test whether the type of alcohol modifies effectiveness, and to isolate whether benefits reflect alcohol in general or the particular alcohol (EtOH vs IPA), enhancing generalizability across practice settings.

Two reviewers, or three if consensus was not reached, independently assessed risk of bias of all studies using the RoB2 tool for randomized studies, assessing potential bias due to the randomization method, protocol deviations, missing data, measurement of the outcome, and selection of the reported results (11).

To explore the presence of small-study effects, funnel plots were generated and Egger's linear regression test was performed to assess asymmetry. A non-significant p-value in Egger's test was interpreted as an absence of strong evidence for small-study effects,

© 2026 Drugeon B et al. *JAMA Network Open*.

rather than as definitive evidence against reporting or publication bias (12). Bias due to missing evidence and selective reporting was therefore assessed separately at the network level using the Risk of Bias due to Missing Evidence in Network Meta-analysis (ROB-MEN) framework, which integrates judgments on pairwise comparisons with their contribution to network estimates (13). This approach allows a structured assessment of potential reporting bias by combining judgments on bias in direct pairwise comparisons with information on their relative contribution to each network estimate.

For each outcome, all direct comparisons were first classified according to the risk of bias due to missing evidence (no bias detected, suspected bias favoring CHG-based antiseptics, or suspected bias favoring PVI-based antiseptics), based on the direction and completeness of the available evidence across outcomes. Contribution matrices were then derived from the network meta-analysis to quantify the relative influence of each direct comparison on the corresponding network estimates.

These components were integrated to determine, for each network estimate, the proportion of evidence arising from comparisons with suspected bias and to derive an overall judgment of risk of bias due to missing evidence, following the ROB-MEN guidance. Given the structure of the networks, which were almost exclusively informed by direct comparisons and lacked closed loops generating meaningful indirect evidence, the assessment focused primarily on direct evidence, as recommended for sparse or predominantly direct networks.

REFERENCES

1. Bland M. An Introduction to Medical Statistics. 3rd Edition. Oxford: Oxford University Press; 2000. 422 p.

2. Wan X, Wang W, Liu J, Tong T. Estimating the sample mean and standard deviation from the sample size, median, range and/or interquartile range. BMC Med Res Methodol. 2014;14(1):135.

3. Luo D, Wan X, Liu J, Tong T. Optimally estimating the sample mean from the sample size, median, mid-range, and/or mid-quartile range. Stat Methods Med Res. 2018;27(6):1785–805.

4. J. Sweeting M, J. Sutton A, C. Lambert P. What to add to nothing? Use and avoidance of continuity corrections in meta-analysis of sparse data. Statistics in Medicine. 2004;23(9):1351–75.

5. Ren Y, Lin L, Lian Q, Zou H, Chu H. Real-world Performance of Meta-analysis Methods for Double-Zero-Event Studies with Dichotomous Outcomes Using the Cochrane Database of Systematic Reviews. J GEN INTERN MED. 2019;34(6):960–8.

6. Viechtbauer W. Conducting Meta-Analyses in *R* with the **metafor** Package. J Stat Soft [Internet]. 2010 [cited 2024 Oct 7];36(3). Available from: http://www.jstatsoft.org/v36/i03/

7. Csardi G, Nepusz T. The igraph software package for complex network research. [Internet]. 2006. Available from: https://CRAN.R-project.org/package=igraph

8. Rücker G, Krahn U, Köning J, Efthimiou O, Davies A, Schwarzer G. netmeta: Network meta-analysis using frequentist methods [Internet]. 2021. Available from: https://cran.r-project.org/package=netmeta

9. Higgins JPT. Measuring inconsistency in meta-analyses. BMJ. 2003;327(7414):557–60.

10. Von Hippel PT. The heterogeneity statistic I2 can be biased in small meta-analyses. BMC Med Res Methodol. 2015;15(1):35.

11. Sterne JAC, Savović J, Page MJ, Elbers RG, Blencowe NS, Boutron I, et al. RoB 2: a revised tool for assessing risk of bias in randomised trials. BMJ. 2019;l4898.

12. Egger M, Smith GD, Schneider M, Minder C. Bias in meta-analysis detected by a simple, graphical test. BMJ. 1997;315(7109):629–34.

13. Chiocchia V, Nikolakopoulou A, Higgins JPT, Page MJ, Papakonstantinou T, Cipriani A, Furukawa TA, Siontis GCM, Egger M, Salanti G. ROB-MEN: a tool to assess risk of bias due to missing evidence in network meta-analysis. BMC Med. 2021 Nov

© 2026 Drugeon B et al. *JAMA Network Open*.

23;19(1):304.

eAppendix 4. Assessment of Transitivity and Coherence in the Network Meta-Analysis.

Transitivity

Transitivity was evaluated conceptually by comparing the distribution of the prespecified effect modifiers (catheter type, setting, CHG concentration, and formulation) across CHG–PVI treatment comparisons. These variables showed broadly similar distributions, supporting the assumption of transitivity.

Coherence

The node-splitting analysis was performed to assess the consistency between direct and indirect evidence within the network.

CRBSIs

The results indicate no statistically significant inconsistency across the tested comparisons, as all p-values are greater than 0.05. This supports the overall coherence of the network. Nevertheless, variations in the Ratio of Ratios (RoR) were observed, particularly in comparisons with lower proportions of direct evidence. For instance, the comparison between alcoholic PVI and aqueous CHG (proportion = 0.52) yielded a RoR of 4.84, indicating a notable discrepancy between direct (RR = 2.53) and indirect (RR = 4.84) estimates, though this was not statistically significant (P= .4411). A similar pattern was observed in the comparison between alcoholic PVI and alcoholic CHG, which, despite having a high proportion of direct evidence (0.73), presented a RoR of 1.91 (P= .4411). In contrast, the comparison between aqueous CHG and aqueous PVI (proportion = 0.83) showed closer alignment between direct and indirect effects (RoR = 1.04, P= .4411). Comparisons lacking direct evidence (k = 0), such as aqueous PVI vs alcoholic PVI or aqueous CHG vs alcoholic CHG, could not be evaluated for consistency through node-splitting. Although the absence of statistically significant inconsistency strengthens the internal validity of the network, the variability in RoR values warrants cautious interpretation of comparisons based primarily on indirect evidence.

Global inconsistency assessed with the design-by-treatment interaction model did not show significant disagreement between direct and indirect evidence for CRBSIs (Q = 0.75, df = 1, p = 0.39), supporting overall network consistency. See Table 1 and 2 A4.

Colonizations

The results indicate statistically significant inconsistency for all tested comparisons, as all p-values are below 0.05. This suggests a discrepancy between direct and indirect estimates within the network. Notably, the comparison between alcoholic PVI and alcoholic CHG showed a substantial difference between direct (RR = 6.66) and indirect (RR = 1.47) estimates, with a high RoR of 4.54 and a p-value of 0.0474, indicating significant inconsistency. Similarly, the comparison between aqueous CHG and aqueous PVI revealed a RoR of 2.32 (P= .0474), suggesting disagreement between sources of evidence. Although comparisons such as aqueous CHG vs alcoholic CHG or alcoholic PVI vs aqueous PVI lacked direct evidence (k = 0), precluding formal inconsistency testing, their interpretation should be made cautiously. Overall, despite the network's comprehensiveness, the presence of statistically significant inconsistencies highlights the need for careful interpretation of estimates, especially in comparisons driven by indirect evidence.

For catheter tip colonization, the global inconsistency test indicated significant disagreement between designs (Q = 13.50, df = 1, p = 0.0002), suggesting the presence of global inconsistency in the network.

See Table 3 and 4 A4.

© 2026 Drugeon B et al. *JAMA Network Open*.

Local Infections

Due to the limited number of available comparisons and complete reliance on direct estimates, no formal inconsistency testing could be performed for local infections. All analysed comparisons — alcoholic PVI vs. alcoholic CHG and aqueous PVI vs. alcoholic CHG — had 100% direct evidence (proportion = 1) and lacked indirect counterparts, preventing calculation of the Ratio of Ratios (RoR) or inconsistency p-values. Additionally, the comparison between alcoholic PVI and aqueous PVI relied entirely on indirect estimates (k = 0), further precluding node-splitting analysis. Consequently, although no inconsistency was identified in node-splitting analysis for local infections, this reflects the absence of estimable indirect comparisons rather than confirming the coherence of the network. This limitation arises from the structure of the full network, which includes intra-class comparisons not retained in the focused CHG vs PVI analysis.

For local infections, no evidence of global inconsistency was detected (Q = 1.57, df = 1, p = 0.21). See Table 5 and 6 A4.

Discrepancies

The primary objective of this network meta-analysis was to compare the effectiveness of CHG and PVI to determine the most effective formulation for skin antisepsis. To maintain clinical relevance, comparisons between CHG formulations (e.g., high vs low concentration, aqueous vs alcoholic) and PVI formulations were excluded, focusing only on direct CHG vs PVI comparisons. While this approach aligns with the study's objective, it has notable methodological implications.

Firstly, this selection process results in a reduced and structurally different network, leading to discrepancies between the network meta-analysis effect estimates and the node-splitting analysis results. In the full network, comparisons involving CHG vs CHG or PVI vs PVI contribute indirect evidence, influencing effect estimates through network geometry. By removing these comparisons, the available indirect evidence is altered, which may affect the robustness and precision of the results. The node-splitting analysis, which relies on the full network structure, incorporates comparisons that were omitted in the focused CHG vs PVI network meta-analysis, thereby explaining the observed differences in effect estimates.

Secondly, the exclusion of CHG vs CHG and PVI vs PVI comparisons prevents an assessment of intra-class variability, which could be crucial in understanding whether certain formulations of CHG or PVI differ significantly in efficacy. If, for example, high-concentration alcoholic CHG were considerably more effective than low-concentration aqueous CHG, this would impact the interpretation of CHG vs PVI comparisons. Similarly, differences between alcoholic and aqueous PVI could modify the indirect comparisons within the network. Without these intra-class comparisons, the assumption that all CHG and all PVI formulations share similar efficacy remains untested, which may introduce unmeasured heterogeneity.

Finally, the structural modifications to the network affect the coherence and consistency assessments. The node-splitting analysis evaluates inconsistency between direct and indirect evidence within the full network, including comparisons that were excluded in the CHG vs PVI-focused meta-analysis. This means that the node-splitting results do not perfectly reflect the structure of the reduced network meta-analysis, as they include indirect comparisons influenced by CHG vs CHG and PVI vs PVI studies. As a result, while no significant inconsistency was detected in the node-splitting analysis, this does not necessarily validate the coherence of the reduced CHG vs PVI network.

The decision to exclude CHG vs CHG and PVI vs PVI comparisons ensures clinical relevance but alters the structure of the network meta-analysis and modifies the available indirect evidence. This explains the differences observed between the effect size in the article's network meta-analysis and those obtained from the node-splitting analysis. While this approach remains valid for

© 2026 Drugeon B et al. *JAMA Network Open*.

addressing the study question, it limits the ability to detect intra-class variability and may influence the assessment of network consistency.

© 2026 Drugeon B et al. *JAMA Network Open*.

Table 1 A4. Summary of the node-splitting analysis (CRBSIs)

Comparison	k (Studies)	Proportion Direct Evidence	NMA Effect (RR)	Direct Effect (RR)	Indirect Effect (RR)	Ratio of Ratios (RoR)	P-value
Alcoholic PVI:Alcoholic CHG	2	0.7306	3.6766	4.3780	2.2894	1.9123	.4411
Aqueous CHG:Alcoholic CHG	0	0.0000	1.0624		1.0624		
Aqueous PVI:Alcoholic CHG	8	0.9233	1.7440	1.6595	3.1733	0.5229	.4411
Alcoholic PVI:Aqueous CHG	1	0.5177	3.4607	2.5314	4.8407	0.5229	.4411
Alcoholic PVI:Aqueous PVI	0	0.0000	2.1081		2.1081		
Aqueous CHG:Aqueous PVI	4	0.8284	0.6092	0.5450	1.0422	0.5229	.4411

The column "k (Studies)" indicates the number of studies providing direct evidence for each comparison, while "Proportion Direct Evidence" represents the proportion of evidence derived from direct comparisons relative to the total available data. The "NMA Effect (RR)" corresponds to the estimated Risk Ratio (RR) from the network meta-analysis. The "Direct Effect (RR)" and "Indirect Effect (RR)" columns show the RR values obtained exclusively from direct and indirect evidence, respectively. The "Ratio of Ratios (RoR)" quantifies the relationship between direct and indirect effects, where a RoR ≠ 1 may indicate inconsistency. Finally, the "p-value" column provides the statistical test for inconsistency; a p-value < 0.05 suggests significant inconsistency between direct and indirect estimates.

Table 2 A4. Global inconsistency assessment for CRBSI using the design-by-treatment interaction model

Component	Q	df	p-value
Total	10.46	10	0.40
Within designs	9.71	9	0.37
Between designs	0.75	1	0.39

© 2026 Drugeon B et al. *JAMA Network Open*.

Table 3 A4. Summary of the node-splitting analysis (colonizations)

Comparison	k (Studies)	Proportion Direct Evidence	NMA Effect (RR)	Direct Effect (RR)	Indirect Effect (RR)	Ratio of Ratios (RoR)	P-value
Alcoholic PVI:Alcoholic CHG	3	0.7657	4.6742	6.6625	1.4681	4.5383	.0474
Aqueous CHG:Alcoholic CHG	0	0.0000	1.1036		1.1036		
Aqueous PVI:Alcoholic CHG	8	0.9210	1.6865	1.4966	6.7920	0.2203	.0474
Alcoholic PVI:Aqueous CHG	1	0.4758	4.2355	1.9166	8.6983	0.2203	.0474
Alcoholic PVI:Aqueous PVI	0	0.0000	2.7715		2.7715		
Aqueous CHG:Aqueous PVI	5	0.8376	0.6543	0.5118	2.3228	0.2203	.0474

The column "k (Studies)" indicates the number of studies providing direct evidence for each comparison, while "Proportion Direct Evidence" represents the proportion of evidence derived from direct comparisons relative to the total available data. The "NMA Effect (RR)" corresponds to the estimated Risk Ratio (RR) from the network meta-analysis. The "Direct Effect (RR)" and "Indirect Effect (RR)" columns show the RR values obtained exclusively from direct and indirect evidence, respectively. The "Ratio of Ratios (RoR)" quantifies the relationship between direct and indirect effects, where a RoR ≠ 1 may indicate inconsistency. Finally, the "p-value" column provides the statistical test for inconsistency; a p-value < 0.05 suggests significant inconsistency between direct and indirect estimates.

Table 4 A4. Global inconsistency assessment for catheter tip colonization using the design-by-treatment interaction model

Component	Q	df	p-value
Total	67.05	14	<0.0001
Within designs	53.56	13	<0.0001
Between designs	**13.50**	**1**	**0.0002**

© 2026 Drugeon B et al. *JAMA Network Open*.

Table 5 A4. Summary of the node-splitting analysis (local infections)

Comparison	k (Studies)	Proportion Direct Evidence	NMA Effect (RR)	Direct Effect (RR)	Indirect Effect (RR)	Ratio of Ratios (RoR)	P-value
Alcoholic PVI:Alcoholic CHG	2	1	2.7604	2.7604			
Aqueous PVI:Alcoholic CHG	1	1	8.5304	8.5304			
Alcoholic PVI:Aqueous PVI	0	0	0.3236		0.3236		

The column "k (Studies)" indicates the number of studies providing direct evidence for each comparison, while "Proportion Direct Evidence" represents the proportion of evidence derived from direct comparisons relative to the total available data. The "NMA Effect (RR)" corresponds to the estimated Risk Ratio (RR) from the network meta-analysis. The "Direct Effect (RR)" and "Indirect Effect (RR)" columns show the RR values obtained exclusively from direct and indirect evidence, respectively. The "Ratio of Ratios (RoR)" quantifies the relationship between direct and indirect effects, where a RoR ≠ 1 may indicate inconsistency. Finally, the "p-value" column provides the statistical test for inconsistency; a p-value < 0.05 suggests significant inconsistency between direct and indirect estimates.

Table 6 A4. Global inconsistency assessment for local infections using the design-by-treatment interaction model

Component	Q	df	p-value
Total	1.57	1	0.21
Within designs	1.57	1	0.21
Between designs	—	0	—

© 2026 Drugeon B et al. *JAMA Network Open*.

eAppendix 5. Network plots

Network plots for the association of antiseptic solution type with (A) CRBSI, (B) catheter tip colonization, and (C) local infection. Green circles represent PVI groups, blue circles CHG groups. The size of each circle is proportional to the sample size of the corresponding group. Curves connecting the circles indicate direct comparisons from individual studies, with each curve representing one study.

(A)

(B)

© 2026 Drugeon B et al. *JAMA Network Open*.

(C)

CHG: Chlorhexidine; CRBSI: Catheter-Related Bloodstream Infection; PVI: Povidone-Iodine

© 2026 Drugeon B et al. *JAMA Network Open*.

eAppendix 6. Surface Under the Cumulative Ranking Curve (SUCRA) Analysis

SUCRA and ranking analysis for Antiseptic Strategies in Preventing Catheter-Related Infections, rankograms and league tables for (A) CRBSI, (B) Catheter Tip Colonization, and (C) Local Infection.

Rankograms and SUCRA values summarize the distribution of treatment rankings across the network. They suggested a higher probability for alcoholic CHG to be among the most effective interventions across outcomes, while acknowledging substantial uncertainty. These findings were interpreted cautiously and were not used to support the main conclusions, which relied on relative treatment effects.

(A)

SUCRA, PrBEST & Mean Rank des traitements

Treatment	SUCRA (%)	PrBEST (%)	Mean Rank
Alcoholic CHG	98.7	96.2	1.04
Alcoholic PVI	67.9	3.9	1.96
Aqueous CHG	32.5	0.0	3.03
Aqueous PVI	0.9	0.0	3.97

© 2026 Drugeon B et al. *JAMA Network Open*.

League Table of Network Meta-analysis for CRBSIs.

	Alcoholic CHG	Alcoholic PVI	Aqueous CHG	Aqueous PVI
**Alcoholic CHG**		0.21 [0.08; 0.53]	.	0.59 [0.37; 0.95]
**Alcoholic PVI**	0.26 [0.12; 0.57]		2.53 [0.78; 8.22]	.
**Aqueous CHG**	0.93 [0.44; 1.95]	3.56 [1.51; 8.40]		0.53 [0.26; 1.09]
**Aqueous PVI**	0.56 [0.36; 0.88]	2.15 [0.94; 4.92]	0.61 [0.32; 1.16]	

Each cell presents the Risk Ratio (RR) and 95% Confidence Interval (CI) comparing the treatment in the row to the treatment in the column. An RR < 1 favors the treatment listed in the row. A dot (.) indicates comparisons that are redundant or not estimable within the network. All estimates are derived from the random-effects network meta-analysis.

© 2026 Drugeon B et al. *JAMA Network Open*.

(B)

SUCRA, PrBEST & Mean Rank des traitements

Treatment	SUCRA (%)	PrBEST (%)	Mean Rank
Alcoholic CHG	100.0	100	1.00
Alcoholic PVI	45.5	0	2.63
Aqueous CHG	54.5	0	2.37
Aqueous PVI	0.0	0	4.00

League Table of Network Meta-analysis for catheter tip colonizations.

	Alcoholic CHG	Alcoholic PVI	Aqueous CHG	Aqueous PVI
**Alcoholic CHG**		0.15 [0.07; 0.31]	.	0.67 [0.44; 1.02]
**Alcoholic PVI**	0.21 [0.11; 0.40]		1.92 [0.65; 5.66]	.
**Aqueous CHG**	0.91 [0.48; 1.72]	4.24 [2.01; 8.94]		0.51 [0.28; 0.93]
**Aqueous PVI**	0.59 [0.40; 0.89]	2.77 [1.39; 5.54]	0.65 [0.38; 1.14]	

Each cell presents the Risk Ratio (RR) and 95% Confidence Interval (CI) comparing the treatment in the row to the treatment in the column. An RR < 1 favors the treatment listed in the row. A dot (.) indicates comparisons that are

redundant or not estimable within the network. All estimates are derived from the random-effects network meta-analysis.

© 2026 Drugeon B et al. *JAMA Network Open*.

(C)

SUCRA, PrBEST & Mean Rank des traitements

Treatment	SUCRA (%)	PrBEST (%)	Mean Rank
Alcoholic CHG	99.6	99.3	1.01
Alcoholic PVI	39.5	0.1	2.21
Aqueous PVI	10.9	0.6	2.78

League Table of Network Meta-analysis for local infections.

	Alcoholic CHG	Alcoholic PVI	Aqueous PVI
**Alcoholic CHG**		0.34 [0.08; 1.50]	0.10 [0.00; 2.85]
**Alcoholic PVI**	0.34 [0.08; 1.50]		.
**Aqueous PVI**	0.10 [0.00; 2.85]	0.31 [0.01; 11.58]	

Each cell presents the Risk Ratio (RR) and 95% Confidence Interval (CI) comparing the treatment in the row to the treatment in the column. An RR < 1 favors the treatment listed in the row. A dot (.) indicates comparisons that are redundant or not estimable within the network. All estimates are derived from the random-effects network meta-analysis.

© 2026 Drugeon B et al. *JAMA Network Open*.

CHG: Chlorhexidine; CRBSI: Catheter-Related Bloodstream Infection; PVI: Povidone-Iodine; SUCRA: Surface under the cumulative ranking

© 2026 Drugeon B et al. *JAMA Network Open*.

eAppendix 7. Sensitivity analyses

Sensitivity analyses excluding high-risk of bias studies confirm the superiority of alcoholic CHG over the others antiseptics solutions in reducing CRBSIs (nine studies) (1–9), catheters tip colonization (ten studies) (1,2,4–11), and local infections (two studies) (2,8). Aqueous CHG was associated with lower CRBSI rates compared with aqueous PVI, and with rates similar to alcoholic PVI. For catheter tip colonization, estimates favored aqueous CHG over both PVI formulations.

eForest Plot 1 A7. Sensitivity analysis of the network meta-analysis of the association between antiseptic solutions and catheter-related infectious complications after exclusion of high-risk of bias studies.

CHG: Chlorhexidine; CRBSI: Catheter-Related Bloodstream Infection; PVI: Povidone Iodine

Conventional pairwise meta-analyses showed lower event rates with CHG-based solutions compared with PVI-based solutions for CRBSIs (RR 0.49, 95% CI 0.35 to 0.68), catheter tip colonization (RR 0.52, 95% CI 0.47 to 0.57), and local infections (RR 0.44, 95% CI 0.25 to 0.77). These estimates, derived from direct evidence alone, were consistent with the results of the network meta-analysis.

© 2026 Drugeon B et al. *JAMA Network Open*.

eForest Plot 2 A7. Pairwise meta-analyses: chlorhexidine vs povidone-iodine for CRBSI, catheter-tip colonization, and local infection.

© 2026 Drugeon B et al. *JAMA Network Open*.

REFERENCES

1. Garland JS, Alex CP, Uhing MR, Peterside IE, Rentz A, Harris MC. Pilot trial to compare tolerance of chlorhexidine gluconate to povidone-iodine antisepsis for central venous catheter placement in neonates. J Perinatol. 2009;29(12):808–13.

2. Guenezan J, Marjanovic N, Drugeon B, Neill RO, Liuu E, Roblot F, et al. Chlorhexidine plus alcohol versus povidone iodine plus alcohol, combined or not with innovative devices, for prevention of short-term peripheral venous catheter infection and failure (CLEAN 3 study): an investigator-initiated, open-label, single centre, randomised-controlled, two-by-two factorial trial. Lancet Infect Dis. 2021;21(7):1038–48.

3. Kieran EA, O'Sullivan A, Miletin J, Twomey AR, Knowles SJ, O'Donnell CPF. 2% chlorhexidine–70% isopropyl alcohol versus 10% povidone–iodine for insertion site cleaning before central line insertion in preterm infants: a randomised trial. Arch Dis Child Fetal Neonatal Ed. 2018;103(2):F101–6.

4. Legras A, Cattier B, Boulain T, Perrotin D. Etude prospective randomis6e pour la prevention des infections li es aux catheters : chlorhexidine alcoolique contre polyvidone iod e. Réanimation Urgences. 1997;6(1):5–11.

5. Maki DG, Alvarado CJ, Ringer M. Prospective randomised trial of povidone-iodine, alcohol, and chlorhexidine for prevention of infection associated with central venous and arterial catheters. The Lancet. 1991;338(8763):339–43.

6. Mimoz O, Pieroni L, Lawrence C, Edouard A, Costa Y, Samii K, et al. Prospective, randomized trial of two antiseptic solutions for prevention of central venous or arterial colonization and infection in intensive care unit patients. Critical Care Medicine. 1996;24(11):1818–23.

7. Mimoz O, Villeminey S, Ragot S, Dahyot-Fizelier C, Laksiri L, Petitpas F, et al. Chlorhexidine-Based Antiseptic Solution vs Alcohol-Based Povidone-Iodine for Central Venous Catheter Care. Archives of Internal Medicine. 2007;167(19):2066.

8. Mimoz O, Lucet JC, Kerforne T, Pascal J, Souweine B, Goudet V, et al. Skin antisepsis with chlorhexidine–alcohol versus povidone iodine–alcohol, with and without skin scrubbing, for prevention of intravascular-catheter-related infection (CLEAN): an open-label, multicentre, randomised, controlled, two-by-two factorial trial. The Lancet. 2015;386(10008):2069–77.

9. Yasuda H, Sanui M, Abe T, Shime N, Komuro T, Hatakeyama J, et al. Comparison of the efficacy of three topical antiseptic solutions for the prevention of catheter colonization: a multicenter randomized controlled study. Crit Care. 2017;21(1):320.

10. Cobbett S, LeBlanc A. Minimising IV site infection while saving time and money. Australian Infection Control. 2000;5(2):8–14.

11. Langgartner J, Linde HJ, Lehn N, Reng M, Scholmerich J, Glück T. Combined skin disinfection with chlorhexidine/propanol and aqueous povidone-iodine reduces bacterial colonisation of central venous catheters. Intensive Care Medicine. 2004;30(6):1081–8.

© 2026 Drugeon B et al. *JAMA Network Open*.

eAppendix 8. Secondary outcome analysis and Subgroup analysis.

Secondary outcome analysis : Lenght of stay (LOS)

Five studies investigated the impact of antiseptic solution choice on LOS (40,42,43,47,49). Alcoholic CHG was associated with longer LOS compared with alcoholic PVI and shorter LOS compared to aqueous PVI. Heterogeneity between studies was substantial (I^2 99.3%, 95% CI 98.7 to 99.6%).

eForest Plot 1 A8. Forest plots for network meta-analysis antiseptic solutions and Hospital Length of Stay (days).

Subgroup analysis

Across subgroup analyses restricted to adult patients (14 studies) (1–14), central venous catheters (12 studies) (1–10,15,16), and peripheral intravenous catheters (two studies) (11,12), effect estimates consistently favored alcoholic CHG over aqueous or alcoholic PVI formulations for CRBSIs, catheter tip colonization, and local infections. In these analyses, aqueous CHG was associated with lower event rates than aqueous PVI and yielded estimates similar to those observed with alcoholic PVI, except for CRBSIs, for which estimates favored alcoholic PVI. In infants, evidence was limited to two studies comparing aqueous or alcoholic CHG with aqueous PVI; effect estimates for CRBSIs and catheter tip colonization were comparable across interventions (15,16).

© 2026 Drugeon B et al. *JAMA Network Open*.

eForest Plot 2 A8. Subgroup analysis of the network meta-analysis of the association between antiseptic solutions and (A) CRBSIs, (B) catheter tip colonization, (C) local infections according to patient age group (adults and infants) and catheter type (CVCs and PIVCs), and (D) decade of study publication.

(A)

© 2026 Drugeon B et al. *JAMA Network Open*.

(B)

(C)

© 2026 Drugeon B et al. *JAMA Network Open*.

(D)

CHG: Chlorhexidine; CRBSI: Catheter-Related Bloodstream Infection; CVC: Central Venous Catheter, PIVC: Peripheral intravenous Catheter; PVI: Povidone Iodine

REFERENCES

1. Atahan K, Cokmez A, Bekoglu M, Durak E, Tavusbay C, Tarcan E. The effect of antiseptic solution in central venous catheter care. BLL. 2012;113(09):548–51.

2. Humar A, Ostromecki A, Direnfeld J, Marshall JC, Lazar N, Houston PC, et al. Prospective Randomized Trial of 10% Povidone-Iodine versus 0.5% Tincture of Chlorhexidine as Cutaneous Antisepsis for Prevention of Central Venous Catheter Infection. CLIN INFECT DIS. 2000;31(4):1001–7.

3. Legras A, Cattier B, Boulain T, Perrotin D. Etude prospective randomis6e pour la prevention des infections li es aux catheters : chlorhexidine alcoolique contre polyvidone iod e. Réanimation Urgences. 1997;6(1):5–11.

4. Maki DG, Alvarado CJ, Ringer M. Prospective randomised trial of povidone-iodine, alcohol, and chlorhexidine for prevention of infection associated with central venous and arterial catheters. The Lancet. 1991;338(8763):339–43.

5. Maki DG, knasinski V, Narans LL, Gordon BJ. A Randomized Trial ofa Novel 1% Chlorhexidine-75% Alcohol Tincture vs. 10% Povidone- Iodine for Cutaneous disinfection with Vascular Catheters. Society for Healthcare Epidemiology of America. 2001;

6. Mimoz O, Pieroni L, Lawrence C, Edouard A, Costa Y, Samii K, et al. Prospective, randomized trial of two antiseptic solutions for prevention of central venous or arterial colonization and infection in intensive care unit patients. Critical Care Medicine. 1996;24(11):1818–23.

7. Mimoz O, Villeminey S, Ragot S, Dahyot-Fizelier C, Laksiri L, Petitpas F, et al. Chlorhexidine-Based Antiseptic Solution vs Alcohol-Based Povidone-Iodine for Central Venous Catheter Care. Archives of Internal Medicine. 2007;167(19):2066.

8. Mimoz O, Lucet JC, Kerforne T, Pascal J, Souweine B, Goudet V, et al. Skin antisepsis with chlorhexidine–alcohol versus povidone iodine–alcohol, with and without skin scrubbing, for prevention of intravascular-catheter-related infection (CLEAN): an open-label, multicentre, randomised, controlled, two-by-two factorial trial. The Lancet. 2015;386(10008):2069–77.

© 2026 Drugeon B et al. *JAMA Network Open*.

9. Vallés J, Fernández I, Alcaraz D, Chacón E, Cazorla A, Canals M, et al. Prospective Randomized Trial of 3 Antiseptic Solutions for Prevention of Catheter Colonization in an Intensive Care Unit for Adult Patients. Infect Control Hosp Epidemiol. 2008;29(9):847–53.

10. Yasuda H, Sanui M, Abe T, Shime N, Komuro T, Hatakeyama J, et al. Comparison of the efficacy of three topical antiseptic solutions for the prevention of catheter colonization: a multicenter randomized controlled study. Crit Care. 2017;21(1):320.

11. Cobbett S, LeBlanc A. Minimising IV site infection while saving time and money. Australian Infection Control. 2000;5(2):8–14.

12. Guenezan J, Marjanovic N, Drugeon B, Neill RO, Liuu E, Roblot F, et al. Chlorhexidine plus alcohol versus povidone iodine plus alcohol, combined or not with innovative devices, for prevention of short-term peripheral venous catheter infection and failure (CLEAN 3 study): an investigator-initiated, open-label, single centre, randomised-controlled, two-by-two factorial trial. Lancet Infect Dis. 2021;21(7):1038–48.

13. Langgartner J, Linde HJ, Lehn N, Reng M, Scholmerich J, Glück T. Combined skin disinfection with chlorhexidine/propanol and aqueous povidone-iodine reduces bacterial colonisation of central venous catheters. Intensive Care Medicine. 2004;30(6):1081–8.

14. Sheehan G, Leicht K, O'Brien M, Taylor G, Rennie R. Chlorhexidine versus Povidone-Iodine as cutaneous atisepsis for prevention of vascular-cathter infection. Interscience Conference of Antimicrobial Agents and Chemotherapy. Abstract n°1616:1993.

15. Garland JS, Alex CP, Uhing MR, Peterside IE, Rentz A, Harris MC. Pilot trial to compare tolerance of chlorhexidine gluconate to povidone-iodine antisepsis for central venous catheter placement in neonates. J Perinatol. 2009;29(12):808–13.

16. Kieran EA, O'Sullivan A, Miletin J, Twomey AR, Knowles SJ, O'Donnell CPF. 2% chlorhexidine–70% isopropyl alcohol versus 10% povidone–iodine for insertion site cleaning before central line insertion in preterm infants: a randomised trial. Arch Dis Child Fetal Neonatal Ed. 2018;103(2):F101–6.

© 2026 Drugeon B et al. *JAMA Network Open*.

eAppendix 9. Assessment of risk of bias and reporting bias.

RISK OF STUDIES BIAS

A summary of risk of bias in included studies is provided in Figure 1 A9. Randomization method, deviation from planned interventions and missing outcome data were items most frequently identified as of high risk of bias.

Randomization, blinding and concealment

Nine studies did not describe their randomization method (1–9), five used numbered, sealed, opaque envelopes (10–14), and two used a secure web-based centralized randomization system (15,16).

Protocol deviations and missing data

One study reported limited sample size, failing to detect significant difference between antiseptics (1). Two short-papers lacked details, raising concerns about high bias risk from protocol deviations and missing data handling. Uncertainty over whether all randomized patients were analyzed suggests lack of true ITT analysis. Missing follow-up details and possible exclusions increase attrition bias, limiting confidence in the study validity (7,8). Two studies presented high risk of attrition bias due to significant post-randomization exclusions and limitations in ITT analysis. In one study, 35% of patients were excluded from the primary analysis because their catheter was removed or they died within 72 hours post-insertion. As a result, the primary analysis was conducted on 242 patients who retained their catheter for ≥72 hours. While an ITT analysis was performed to include these excluded patients, the results remained unchanged from the primary analysis (4). Similarly, another study, which included 998 catheters, excluded 279 (28%) for not meeting criteria and 88 (9%) for prolonged use, introducing attrition bias. The primary analysis was conducted on evaluable catheters, and while a modified ITT analysis was performed using a worst-case imputation, assigning catheter tip colonization to excluded cases, this approach did not fully restore the original randomization or eliminate bias (9). Additionally, lack of details on protocol deviations further limits the reliability of the findings.

Measurement of the outcome

Most of the included studies appropriately measure interrelation outcomes. However, one study, in addition to performing catheters tip culture, generally considered the standard for determining colonization or CRBSI, to also cultured the catheters hub (7).

Selection of the reported results

None of the selected studies had a high risk of bias regarding outcome reporting selection, as the chosen infection criteria did not allow for multiple interpretations of the results.

© 2026 Drugeon B et al. *JAMA Network Open*.

eFigure 1 A9. Summary plot of study-level risk of bias across RoB 2 domains. Graph (A) and summary (B): review authors' judgment about each risk of bias item.

(A)

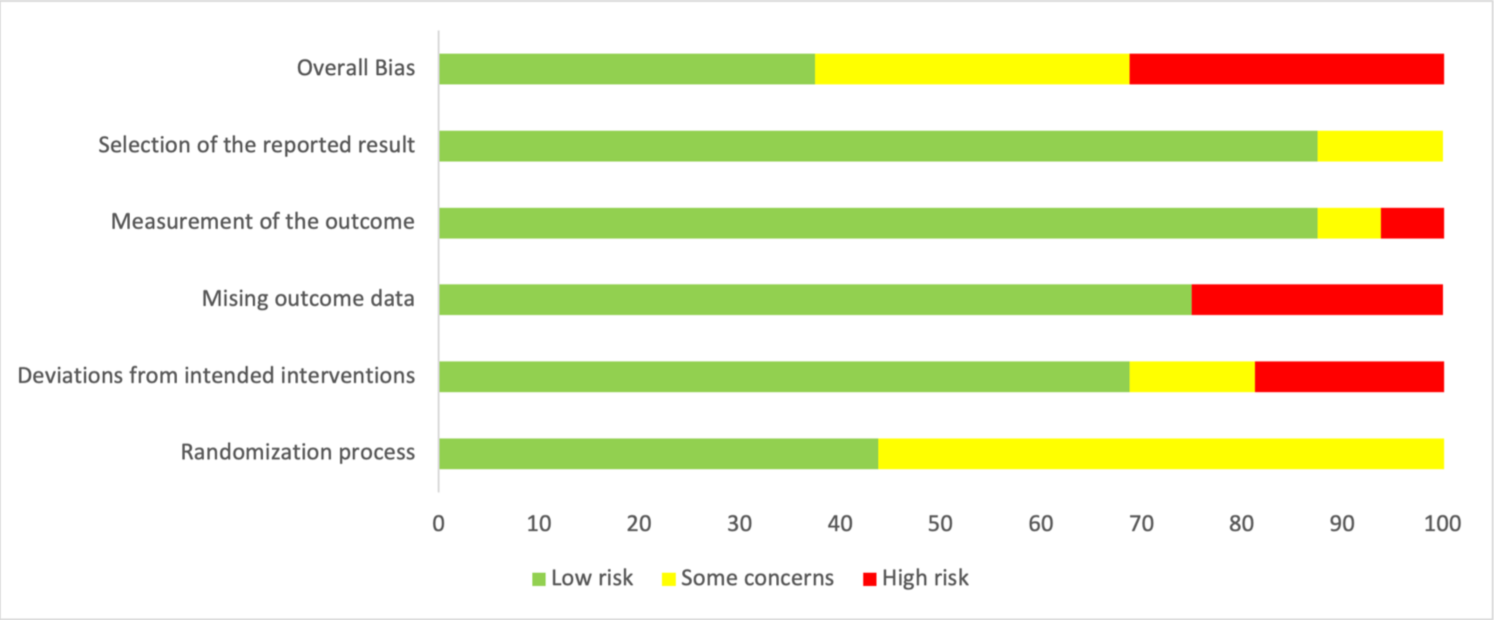

(B)

© 2026 Drugeon B et al. *JAMA Network Open*.

ASSESSMENT OF REPORTING BIASES

For catheters tip colonization, the test did not reveal significant asymmetry (t=−0.74, P= .70), suggesting no strong evidence of small-study effects. Similarly, for CRBSI, although the bias estimate was larger, the p-value remained non-significant (t=−1.05, P= .32), again indicating no clear evidence of small-study effects.

As funnel plots and Egger's tests assess small-study effects rather than publication or reporting bias per se, these analyses were interpreted cautiously and were not used as definitive indicators of bias due to missing evidence. Instead, potential reporting bias was evaluated at the network level using the ROB-MEN framework (17) (Table 1 A9), which integrates judgments on pairwise comparisons with their contribution to network estimates.

Owing to the limited number of studies contributing to the local infection outcome (<10 studies), formal assessment of small-study effects was not performed for this outcome (18) (Figure 2 A9).

Figure 2 A9: Funnel plots illustrates small-study effects for studies evaluating (A) CRBSI and (B) catheter tip colonization comparing CHG versus PVI

(A)

© 2026 Drugeon B et al. *JAMA Network Open*.

(B)

© 2026 Drugeon B et al. *JAMA Network Open*.

Table 1 A9. Network-level assessment of risk of bias due to missing evidence (ROB-MEN) for CRBSI, colonisation, and local infection outcomes

NMA estimate	% contribution of evidence from pairwise comparisons with suspected bias		Evaluation of contribution from evidence with suspected bias	Overall risk of bias
	Favoring CHG	Favoring PVI		
**CRBSI**				
Alcoholic CHG:Alcoholic PVI	0	0	No substantial contribution from bias	Low risk
Alcoholic CHG:Aqueous PVI	0	0	No substantial contribution from bias	Low risk
Alcoholic PVI:Aqueous CHG	0	0	No substantial contribution from bias	Low risk
Aqueous CHG:Aqueous PVI	0	0	No substantial contribution from bias	Low risk
**COLONIZATION**				
Alcoholic CHG:Alcoholic PVI	0	0	No substantial contribution from bias	Low risk
Alcoholic CHG:Aqueous PVI	0	0	No substantial contribution from bias	Low risk
Alcoholic PVI:Aqueous CHG	0	0	No substantial contribution from bias	Low risk
Aqueous CHG:Aqueous PVI	0	0	No substantial contribution from bias	Low risk
**LOCAL INFECTION**				
Alcoholic CHG:Alcoholic PVI	0	0	No substantial contribution from bias	Low risk
Alcoholic CHG:Aqueous PVI	0	0	No substantial contribution from bias	Low risk

CHG: Chlorhexidine; CRBSI: Catheter-Related Bloodstream Infection; NMA: Network Meta Analysis; PVI: Povidone Iodine

For the colonization and local infection outcomes, no meaningful indirect evidence contributed to the network estimates. Consequently, bias due to missing evidence was assessed at the level of direct comparisons only. For CRBSI, all network estimates were entirely informed (100% contribution) by direct comparisons judged at low risk of bias due to missing evidence, with no contribution from comparisons suspected of favouring either CHG-based or PVI-based antiseptics. Accordingly, the risk of bias due to missing evidence was considered low for all network estimates.

REFERENCES

1. Atahan K, Cokmez A, Bekoglu M, Durak E, Tavusbay C, Tarcan E. The effect of antiseptic solution in central venous catheter care. BLL. 2012;113(09):548–51.

© 2026 Drugeon B et al. *JAMA Network Open*.

2. Cobbett S, LeBlanc A. Minimising IV site infection while saving time and money. Australian Infection Control. 2000;5(2):8–14.

3. Garland JS, Alex CP, Uhing MR, Peterside IE, Rentz A, Harris MC. Pilot trial to compare tolerance of chlorhexidine gluconate to povidone-iodine antisepsis for central venous catheter placement in neonates. J Perinatol. 2009;29(12):808–13.

4. Humar A, Ostromecki A, Direnfeld J, Marshall JC, Lazar N, Houston PC, et al. Prospective Randomized Trial of 10% Povidone-Iodine versus 0.5% Tincture of Chlorhexidine as Cutaneous Antisepsis for Prevention of Central Venous Catheter Infection. CLIN INFECT DIS. 2000;31(4):1001–7.

5. Legras A, Cattier B, Boulain T, Perrotin D. Etude prospective randomis6e pour la prevention des infections li es aux catheters : chlorhexidine alcoolique contre polyvidone iod e. Réanimation Urgences. 1997;6(1):5–11.

6. Maki DG, Alvarado CJ, Ringer M. Prospective randomised trial of povidone-iodine, alcohol, and chlorhexidine for prevention of infection associated with central venous and arterial catheters. The Lancet. 1991;338(8763):339–43.

7. Maki DG, knasinski V, Narans LL, Gordon BJ. A Randomized Trial ofa Novel 1% Chlorhexidine-75% Alcohol Tincture vs. 10% Povidone- Iodine for Cutaneous disinfection with Vascular Catheters. Society for Healthcare Epidemiology of America. 2001;

8. Sheehan G, Leicht K, O'Brien M, Taylor G, Rennie R. Chlorhexidine versus Povidone-Iodine as cutaneous atisepsis for prevention of vascular-cathter infection. Interscience Conference of Antimicrobial Agents and Chemotherapy. Abstract n°1616:1993.

9. Vallés J, Fernández I, Alcaraz D, Chacón E, Cazorla A, Canals M, et al. Prospective Randomized Trial of 3 Antiseptic Solutions for Prevention of Catheter Colonization in an Intensive Care Unit for Adult Patients. Infect Control Hosp Epidemiol. 2008;29(9):847–53.

10. Mimoz O, Pieroni L, Lawrence C, Edouard A, Costa Y, Samii K, et al. Prospective, randomized trial of two antiseptic solutions for prevention of central venous or arterial colonization and infection in intensive care unit patients. Critical Care Medicine. 1996;24(11):1818–23.

11. Mimoz O, Villeminey S, Ragot S, Dahyot-Fizelier C, Laksiri L, Petitpas F, et al. Chlorhexidine-Based Antiseptic Solution vs Alcohol-Based Povidone-Iodine for Central Venous Catheter Care. Arch Intern Med. 2007;167(19):2066.

12. Langgartner J, Linde HJ, Lehn N, Reng M, Scholmerich J, Glück T. Combined skin disinfection with chlorhexidine/propanol and aqueous povidone-iodine reduces bacterial colonisation of central venous catheters. Intensive Care Medicine. 2004;30(6):1081–8.

13. Yasuda H, Sanui M, Abe T, Shime N, Komuro T, Hatakeyama J, et al. Comparison of the efficacy of three topical antiseptic solutions for the prevention of catheter colonization: a multicenter randomized controlled study. Crit Care. 2017;21(1):320.

14. Kieran EA, O'Sullivan A, Miletin J, Twomey AR, Knowles SJ, O'Donnell CPF. 2% chlorhexidine–70% isopropyl alcohol versus 10% povidone–iodine for insertion site cleaning before central line insertion in preterm infants: a randomised trial. Arch Dis Child Fetal Neonatal Ed. 2018;103(2):F101–6.

15. Guenezan J, Marjanovic N, Drugeon B, Neill RO, Liuu E, Roblot F, et al. Chlorhexidine plus alcohol versus povidone iodine plus alcohol, combined or not with innovative devices, for prevention of short-term peripheral venous catheter infection and failure (CLEAN 3 study): an investigator-initiated, open-label, single centre, randomised-controlled, two-by-two factorial trial. Lancet Infect Dis. 2021;21(7):1038–48.

16. Mimoz O, Lucet JC, Kerforne T, Pascal J, Souweine B, Goudet V, et al. Skin antisepsis with chlorhexidine–alcohol versus povidone iodine–alcohol, with and without skin scrubbing, for prevention of intravascular-catheter-related infection (CLEAN): an open-label, multicentre, randomised, controlled, two-by-two factorial trial. The Lancet. 2015;386(10008):2069–77.

17. Chiocchia V, Nikolakopoulou A, Higgins JPT, Page MJ, Papakonstantinou T, Cipriani A, Furukawa TA, Siontis GCM, Egger M, Salanti G. ROB-MEN: a tool to assess risk of bias due to missing evidence in network meta-analysis. BMC Med. 2021 Nov 23;19(1):304.

© 2026 Drugeon B et al. *JAMA Network Open*.

18. Chapter 13: Assessing risk of bias due to missing evidence in a meta-analysis [Internet]. [cited 2024 Oct 28]. Available from: https://training.cochrane.org/handbook/current/chapter-13

© 2026 Drugeon B et al. *JAMA Network Open*.
