# Supplementary material for: Chlorhexidine vs Povidone-Iodine and Incidence of Catheter-Related Infections: A Systematic Review and Meta-Analysis
Source: JAMA Netw Open. 2026 Feb 12;9(2):e2558954. doi: 10.1001/jamanetworkopen.2025.58954 (PMC12902894; doi:10.1001/jamanetworkopen.2025.58954)
Supplement: Supplement 2. — Data Sharing Statement [file jamanetwopen-e2558954-s002.pdf]

Data Sharing Statement

Drugeon. Chlorhexidine vs Povidone-Iodine and Incidence of Catheter-Related Infections. *JAMA Netw Open*. Published February 12, 2026. doi:10.1001/jamanetworkopen.2025.58954

Data
Data available: Yes
Data types: Data (not involving human participants)
How to access data: Bertrand.DRUGEON@chu-poitiers.fr
When available: With publication

Supporting Documents
Document types: None

Additional Information
Who can access the data: The datasets generated and analyzed during the current study, including template data collection forms, data extracted from the included studies, data used for all analyses, analytic code, and any other materials used in the review, are not publicly available but can be provided by the corresponding author upon reasonable request. Researchers interested in accessing these materials for further analysis or validation purposes are encouraged to contact the corresponding author directly.
Types of analyses: further analysis or validation purposes
Mechanisms of data availability: Data will be made available upon reasonable request, with investigator support and following a signed data access agreement.
